# From smart sensing to adaptive remediation: artificial intelligence and Internet of Things for persistent organic pollutants

**DOI:** 10.1007/s10661-026-15975-x

**Published:** 2026-10-14

**Authors:** Khaled Chawraba, Abrar Hussain, Obaid A. Alharbi, Amal Abdelhaleem

**Affiliations:** 1 Department of Civil and Environmental Engineering, The Hong Kong Polytechnic University, Hung Hom, Hong Kong; 2 Advanced Radiation Technology Institute (ARTI), Korea Atomic Energy Research Institute, Jeongeup, 56212 Republic of Korea; 3 Department of Radiation Science, University of Science and Technology, Daejeon, 34113 Republic of Korea; 4 Institute of Water Management and Treatment Technologies, Sustainability and Environment Sector, King Abdulaziz City for Science and Technology (KACST), 11442 Riyadh, Saudi Arabia; 5 Environmental Engineering Department, Egypt-Japan University of Science and Technology, Alexandria, 21934 Egypt

**Keywords:** Adaptive remediation, Closed-loop environmental management, Digital twins, Explainable AI, Predictive analytics

## Abstract

Persistent organic pollutants (POPs) remain a major environmental challenge because of their persistence, long-range transport, bioaccumulation, and resistance to degradation. Conventional monitoring and remediation systems provide reliable analytical performance but are largely fragmented, laboratory dependent, and unable to deliver the continuous, predictive, and adaptive management required for complex, dynamic contamination scenarios. Recent literature reports substantial advances in biosensors, Internet of Things (IoT)-enabled sensing, machine learning (ML), and artificial intelligence (AI) for pollutant detection, forecasting, source identification, and remediation optimization. However, these developments often operate separately, with limited coordination between environmental sensing, data analytics, treatment processes, and decision-making. The literature further highlights persistent barriers to field deployment, including limited and geographically heterogeneous datasets, inconsistent sensor performance and calibration, restricted model transferability, insufficient independent validation, uncertainty in AI predictions, and the lack of mature regulatory frameworks for AI-assisted environmental assessment. From our perspective, the next advance in POP management should move beyond isolated AI applications toward integrated, closed-loop AI–IoT architectures that continuously connect sensing, prediction, remediation, and feedback control. Such systems could transform POP management from reactive assessment to proactive and adaptive environmental governance. Future progress will depend on standardized, Findable, Accessible, Interoperable, and Reusable (FAIR) environmental datasets, explainable and uncertainty-aware AI, robust field validation, and interoperable sensing infrastructures.

## Introduction

**Fig. 1:**
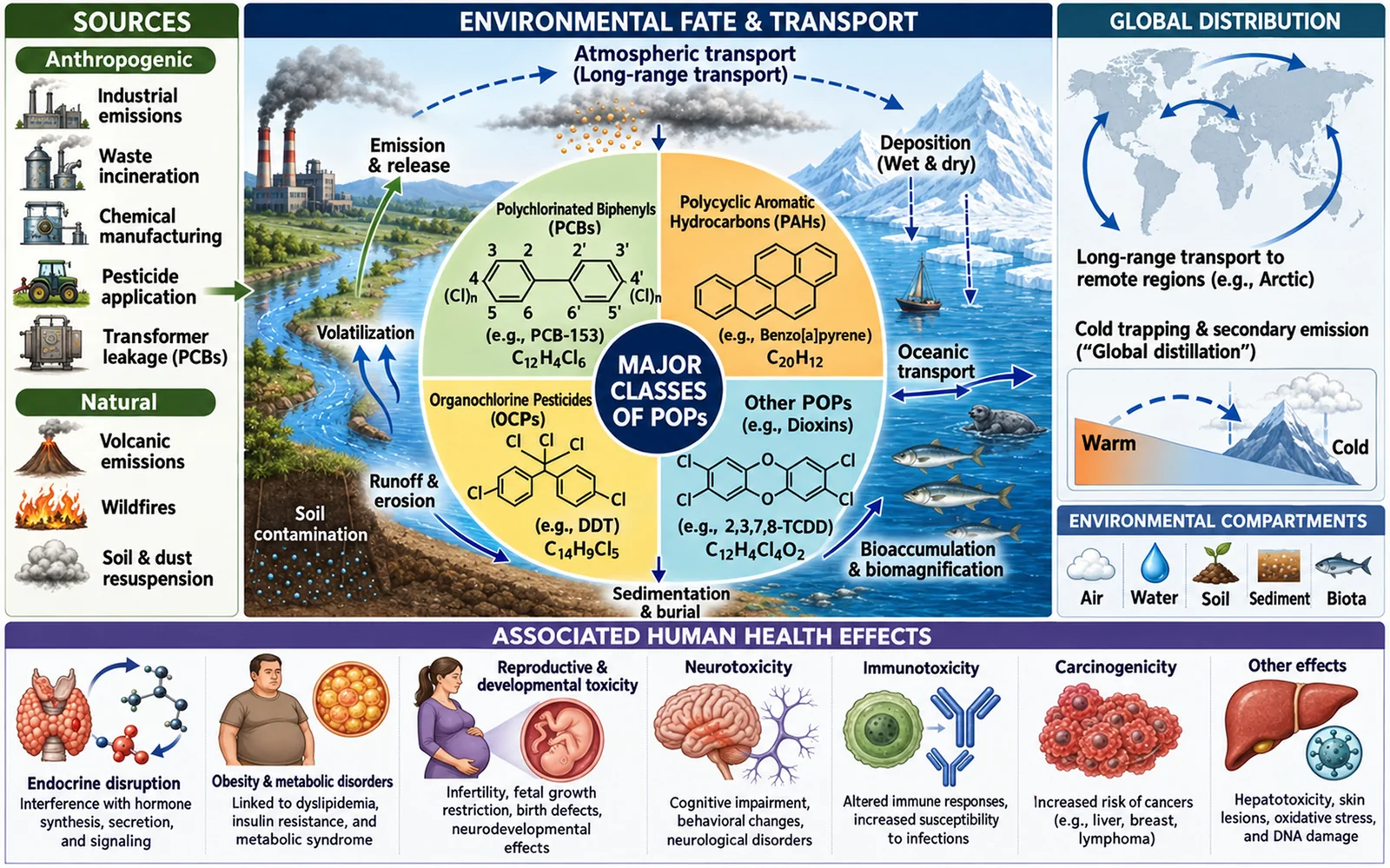
Overview of the major classes of POPs, their environmental fate and transport processes, and associated human health effects

Persistent organic pollutants (POPs) are among the most enduring environmental legacies of industrial development. Unlike conventional contaminants that degrade after release, POPs, including polychlorinated biphenyls (PCBs), organochlorine pesticides (OCPs), and certain brominated flame retardants and per- and polyfluoroalkyl substances (PFAS), along with other persistent organic contaminants such as polycyclic aromatic hydrocarbons (PAHs), persist in the environment for decades because of their exceptional chemical stability, resistance to degradation, and potential for bioaccumulation (National Research Council 2010). Their persistence is further amplified by long-range atmospheric transport and biomagnification, enabling these compounds to spread far beyond their original sources and accumulate throughout the food chain (National Research Council 2010). As illustrated in Fig. 1, POPs continuously cycle among air, water, soil, sediments, plants, animals, and humans, creating a persistent exposure pathway that threatens both ecosystem integrity and public health. The consequences of this persistence extend well beyond environmental contamination. A growing body of epidemiological and toxicological evidence has linked chronic POP exposure to endocrine disruption, carcinogenesis, reproductive impairment, neurodevelopmental disorders, immunotoxicity, metabolic diseases, and biodiversity loss (Pan et al., 2025; Yesildemir & Celik, 2023; Taylor et al., 2013). Although international agreements, particularly the Stockholm Convention, have successfully reduced the production and use of numerous legacy POPs, the environmental challenge remains unresolved because secondary emissions from contaminated soils and sediments, illegal or exempted applications, newly recognized persistent contaminants, and climate-induced remobilization from glaciers, permafrost, and aquatic sediments continue to introduce POPs into the environment (Rosenfeld & Feng, 2011; Li et al., 2006; Kogevinas, 2001; Kallenborn et al., 2012; Venkatesan & Halden, 2014; Wania, 2003; Minh et al., 2006). This persistent and dynamic nature of POP contamination, therefore, highlights the need for monitoring and remediation approaches capable of addressing contamination across changing environmental conditions.

Current POP monitoring and remediation approaches provide important capabilities but remain largely fragmented. Conventional POP monitoring is predominantly reactive: environmental samples are collected periodically, transported to centralized laboratories, and analyzed using sophisticated chromatographic and spectrometric techniques (Shevlin & Ullah, 2023). Although these methodologies provide excellent sensitivity and reliability, they are labor intensive, time consuming, and expensive (Shevlin & Ullah, 2023), and they are limited to discrete temporal and spatial observations (Wu et al., 2008). In parallel, advances in remediation technologies have expanded options for controlling persistent and emerging organic contaminants. Advanced oxidation, adsorption, electrochemical processes, and bioremediation provide complementary approaches for contaminant removal, but their effectiveness depends strongly on pollutant characteristics and operating conditions (Kamalesh et al., 2024). Biochar-based materials have demonstrated considerable potential for PAH remediation, with surface modification and magnetization identified as effective strategies for enhancing their performance (Yaashikaa et al., 2024). Recent studies further demonstrate the potential of engineered carbon-based nanocomposites for contaminant removal (Kumar et al., 2025), while artificial neural networks (ANNs) have shown strong predictive capability for estimating and optimizing pollutant removal efficiency (Kamalesh et al., 2025). At the same time, recent developments in artificial intelligence (AI), machine learning (ML), Internet of Things (IoT) sensor networks, advanced biosensors, cloud computing, and edge analytics have transformed the technological landscape of environmental monitoring (Ramani et al., 2025; Mutlag et al., 2026). Intelligent sensing systems can now continuously collect environmental information, integrate heterogeneous data streams, identify hidden patterns, forecast pollution trends, detect anomalies in real time, and support autonomous decision-making (Mutlag et al., 2026; Yun et al., 2026). Recent reviews have summarized emerging remediation strategies, including advanced oxidation processes (AOPs), nanotechnology, biological approaches, and AI-assisted treatment optimization (Bhat et al., 2026; Okechukwu et al., 2026; Khatri et al., 2025; Sadiq et al., 2025). Similarly, advances in AI, ML, digital sensing, and IoT platforms have been extensively reviewed for environmental monitoring, highlighting their potential for real-time detection, predictive analysis, and intelligent decision support (Popescu et al., 2024; Garcia et al., 2025; Govindarajan et al., 2025; Das & Rad, 2020). Despite these advances, monitoring, prediction, and remediation generally remain separate technological components, limiting their ability to operate as a coordinated, continuously adaptive system.

**Fig. 2:**
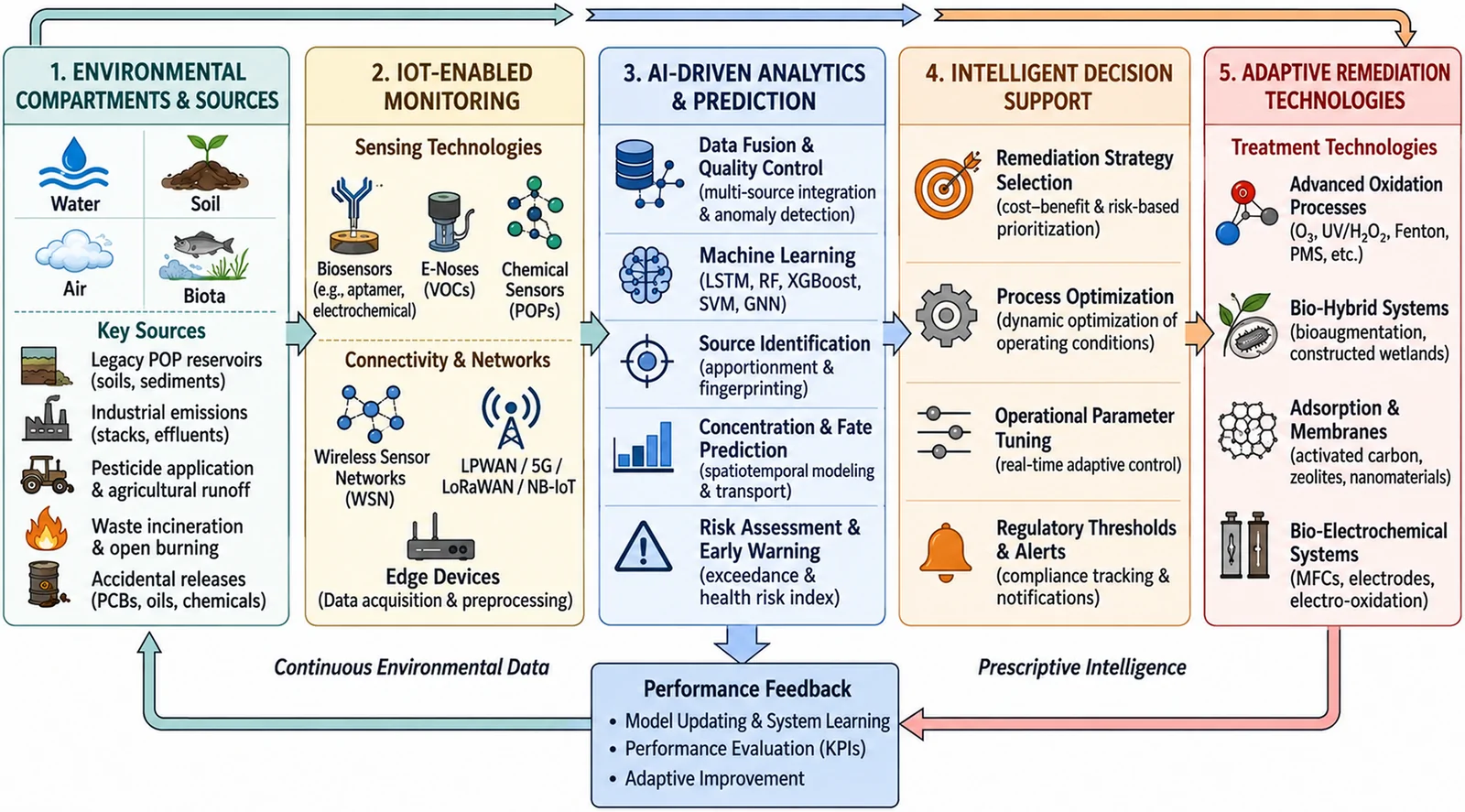
AI–IoT-enabled closed-loop framework linking POP monitoring, prediction, and adaptive remediation

The integration of advanced sensing, AI-driven analytics, predictive modeling, and remediation offers an opportunity to move beyond fragmented monitoring and treatment toward an integrated, adaptive environmental management framework. Recent advances in AI, ML, IoT sensor networks, advanced biosensors, cloud computing, and edge analytics enable continuous collection, integration, and analysis of environmental data to identify hidden patterns, forecast pollution trends, detect anomalies in real time, and support autonomous decision-making (Ramani et al., 2025; Mutlag et al., 2026; Yun et al., 2026). More importantly, these technologies enable direct connections between environmental monitoring and remediation, allowing treatment systems to respond dynamically to changing contamination conditions (Bhat et al., 2026). As illustrated in Fig. 2, IoT-enabled sensor networks and biosensors provide continuous environmental observations that are processed with AI and ML models for data fusion, anomaly detection, pollutant forecasting, and source attribution. The resulting predictive intelligence can then be translated into optimized remediation strategies through AOPs, catalytic treatment systems, adsorption technologies, and hybrid remediation platforms. Treatment performance is fed back into the monitoring system, enabling adaptive learning and autonomous optimization within a closed-loop environmental management architecture. This integration represents a paradigm shift from descriptive environmental monitoring toward predictive, adaptive, and intelligent environmental management, in which sensing, data analysis, remediation, and feedback are continuously connected rather than functioning as independent processes.

Addressing this integration gap is the principal objective and novelty of this review. Unlike previous reviews that primarily evaluate individual technological components, this review provides an integrated, systems-level synthesis that links intelligent sensing, AI-driven analytics, predictive modeling, and adaptive remediation within a unified POP management framework. Rather than treating monitoring and remediation as independent processes, this review examines their dynamic interaction through a closed-loop architecture in which environmental data continuously inform predictive models, remediation decisions, and system optimization. Although recent reviews have demonstrated substantial progress in individual technological domains, including remediation strategies, nanotechnology, biological approaches, AI-assisted treatment optimization, AI, ML, digital sensing, and IoT-based environmental monitoring (Okechukwu et al., 2026; Khatri et al., 2025; Sadiq et al., 2025; Popescu et al., 2024; Garcia et al., 2025; Govindarajan et al., 2025; Das & Rad, 2020), the integration of these components into a unified framework that continuously connects environmental sensing, predictive analytics, remediation, and feedback control remains insufficiently developed. Beyond synthesizing current technological developments, this review critically evaluates the practical challenges that continue to limit the widespread implementation of AI-enabled environmental management, including data scarcity, model generalizability across heterogeneous environmental conditions, interoperability among sensing platforms, explainability and trustworthiness of AI models, cybersecurity of IoT infrastructures, edge computing limitations, and the shortage of large-scale field validation studies. Emerging research directions, including digital twins, federated learning, autonomous environmental sensing, explainable AI, and self-optimizing remediation systems, are also discussed as promising pathways toward next-generation environmental management.

### Methodology of the review

**Fig. 3:**
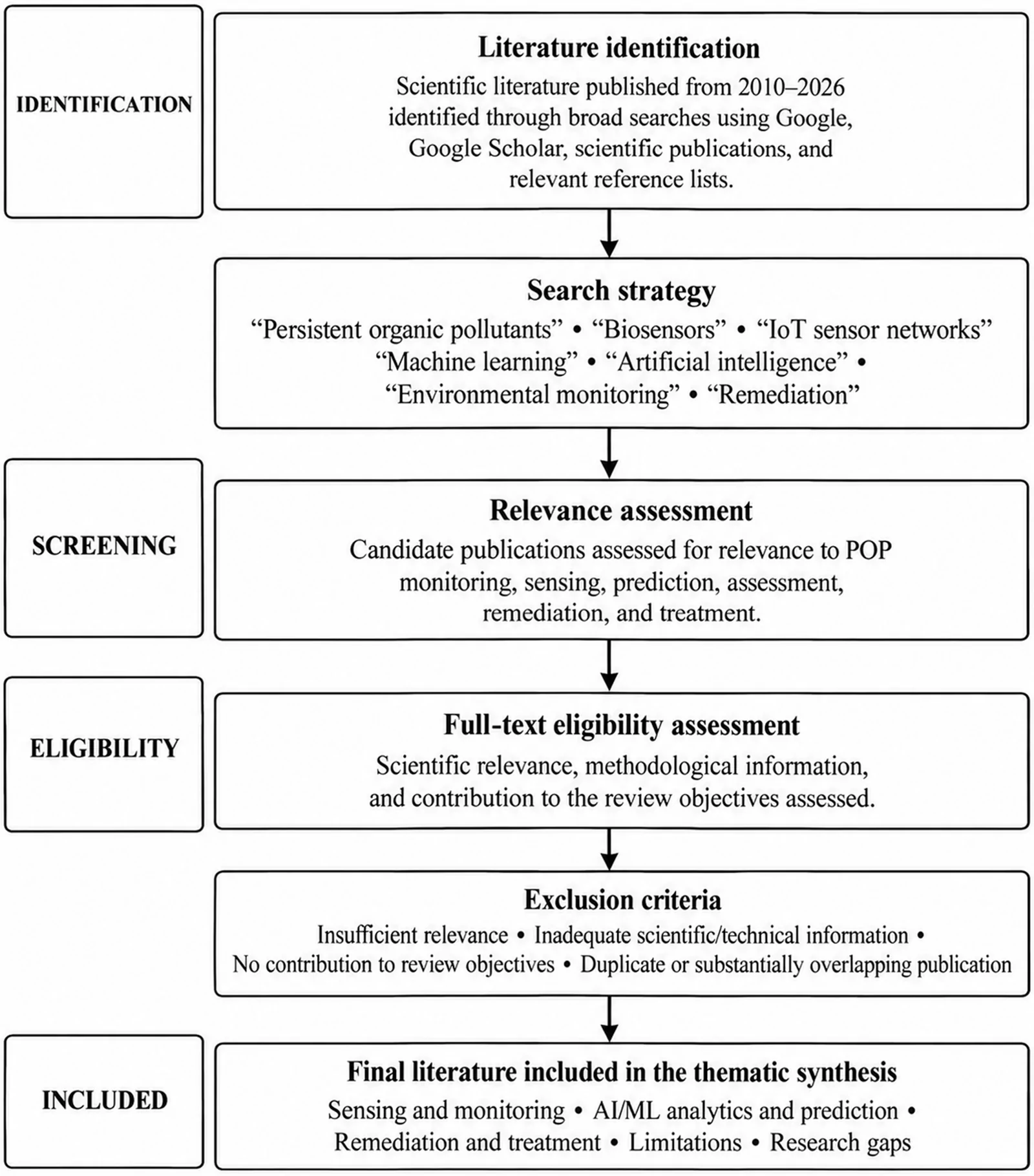
PRISMA-informed literature identification and selection process

This review employed a structured literature review methodology informed by the Preferred Reporting Items for Systematic Reviews and Meta-Analyses (PRISMA) principles to enhance transparency and reproducibility. Relevant scientific literature published from 2010 to 2026 was identified through broad searches, primarily using Google and Google Scholar, supplemented by relevant scientific publications and reference lists. The search combined terms related to “persistent organic pollutants,” “biosensors,” “IoT sensor networks,” “machine learning,” “artificial intelligence,” “environmental monitoring,” and “remediation,” along with related terms and synonyms. The search covered the principal aspects of POP management, including contaminant detection and monitoring, biosensor development, intelligent sensing, AI- and ML-based prediction, IoT-enabled environmental monitoring, environmental assessment, and AI-assisted remediation and treatment. Publications were considered eligible if they addressed POPs or closely related persistent contaminants; concerned environmental monitoring, detection, sensing, prediction, assessment, remediation, or treatment; incorporated AI, ML, IoT, biosensing, sensor networks, or related technologies; or provided relevant scientific context; and contained sufficient scientific or technical information to support the objectives of the review. Publications were excluded if they were insufficiently relevant to POPs or environmental applications, lacked adequate scientific or technical information, did not contribute to the review objectives, or did not support the thematic framework linking sensing, data analytics, prediction, and remediation. Duplicates or substantially overlapping publications were excluded where identified, while selected authoritative institutional and regulatory sources were retained where necessary for regulatory and policy context. The literature-selection process involved identification, relevance assessment, full-text evaluation, and final inclusion in the thematic synthesis. Because the search was conducted as a broad, iterative process using Google/Google Scholar, scientific literature, and reference lists rather than as a prospectively registered systematic review with database-specific retrieval records, the exact numbers of records identified, screened, and excluded were not prospectively recorded; therefore, retrospective numerical PRISMA counts were not estimated. For each publication retained in the final synthesis, information was extracted on the type of POP or contaminant, environmental matrix, sensing and monitoring technology, biosensor configuration, AI/ML method, IoT or communication infrastructure, analytical or predictive function, remediation strategy, reported performance, and principal limitations. The literature was then synthesized across three interconnected domains: (1) sensing and monitoring technologies, (2) AI/ML-based data analytics and prediction, and (3) remediation and treatment processes, enabling examination of how sensing, data acquisition, intelligent analysis, prediction, and remediation can be integrated within an environmental-management framework. The synthesis further identified methodological limitations, technological barriers, research gaps, and opportunities for integrated AI-IoT approaches to POP management. The literature-identification and selection workflow is presented in Fig. 3.

## Monitoring, detection, and regulatory frameworks for POPs

Effective management of POPs rests on two interconnected pillars: (1) robust analytical methodologies capable of detecting trace concentrations across diverse environmental and biological matrices, and (2) governance frameworks that translate monitoring data into regulatory action. This section integrates advances in conventional chemical analysis, biosensing technologies, digital monitoring infrastructures, and mechanisms for international regulatory compliance.

### Analytical methodologies for POPs

Analysis of POPs remains among the most technically demanding tasks in environmental chemistry because of their low environmental concentrations, strong matrix interactions, and structural diversity. Standard workflows include sample preparation, chromatographic separation, and targeted detection, each of which must be optimized to ensure specificity and sensitivity.

#### Sample preparation and extraction

Efficient extraction is essential for isolating POPs from environmental and food matrices. Common approaches include Soxhlet extraction (SE), ultrasonic extraction (UE), accelerated solvent extraction (ASE), and microwave-assisted extraction, all of which aim to maximize recovery, minimize solvent use, and improve cost-effectiveness (Al-Thaiban et al., 2018; Saim et al., 1997). Solvent selection is critical to yield, with dichloromethane (DCM), acetone, and hexane/acetone mixtures frequently used (Bandowe & Meusel, 2017). For complex food matrices, solid-phase microextraction (SPME) and pressurized liquid extraction (PLE) improve analyte selectivity and minimize interference (Muscalu & Górecki, 2018; Ochiai et al., 2011; Xu et al., 2013; El-Shahawi et al., 2010; Wilkowska & Biziuk, 2010).

#### Chromatographic separation and detection

Gas chromatography (GC) remains the benchmark technique for separating semi-volatile, nonpolar POPs such as PCBs and polybrominated diphenyl ethers (PBDEs). Its efficiency depends on analyte volatility and column interactions. However, high-molecular-weight (HMW) PAHs above 300 Da pose challenges due to thermal instability. Two-dimensional gas chromatography (GC × GC) addresses these analytical challenges by providing enhanced resolution through dual-column separation, thereby improving the separation and detection of dioxins and PCBs in complex matrices (L’Homme et al., 2015; Focant et al., 2005).

Liquid chromatography (LC) is highly effective for analyzing polar and thermally labile POPs, including nitro- and oxy-PAHs as well as PFAS. Reversed-phase columns paired with photodiode array (PDA) or fluorescence (FL) detectors help mitigate matrix interference. However, certain analytes, such as oxy-PAHs, may require derivatization (e.g., using *N*,*O*-bis(trimethylsilyl)trifluoroacetamide (BSTFA)) to enhance their volatility and detectability (Shen et al., 2019).

Gas chromatography–mass spectrometry (GC–MS) is the gold standard for POP analysis because of its high sensitivity and specificity. Although electron ionization (EI) is widely used, electron capture negative ionization (ECNI) provides a softer alternative for compounds such as nitro-PAHs and PBDEs, thereby reducing fragmentation (Shen et al., 2019; Poster et al., 2006). High-resolution mass spectrometry (HRMS) and MS/MS configurations enable precise quantification of PCBs and dioxins, particularly when using isotope dilution (L’Homme et al., 2015; Focant et al., 2005; García-Bermejo et al., 2015). LC–MS methods excel at separating and detecting polar POPs such as PFAS. However, atmospheric pressure photoionization (APPI) and electrospray ionization (ESI), particularly when combined with dopants such as acetone, significantly enhance ionization efficiency (Abbas et al., 2018; Ghislain et al., 2012). Alternative detection methods include GC-FID, which is cost-effective for PCBs and OCPs but lacks the selectivity needed to resolve co-eluting compounds (Chung & Chen, 2011; Carabias-Martínez et al., 2005). LC-FL or PDA detectors are suitable for parent PAHs but are limited in their ability to detect polar derivatives without prior derivatization (Lung & Liu, 2015).

#### Analysis in complex matrices

**Fig. 4:**
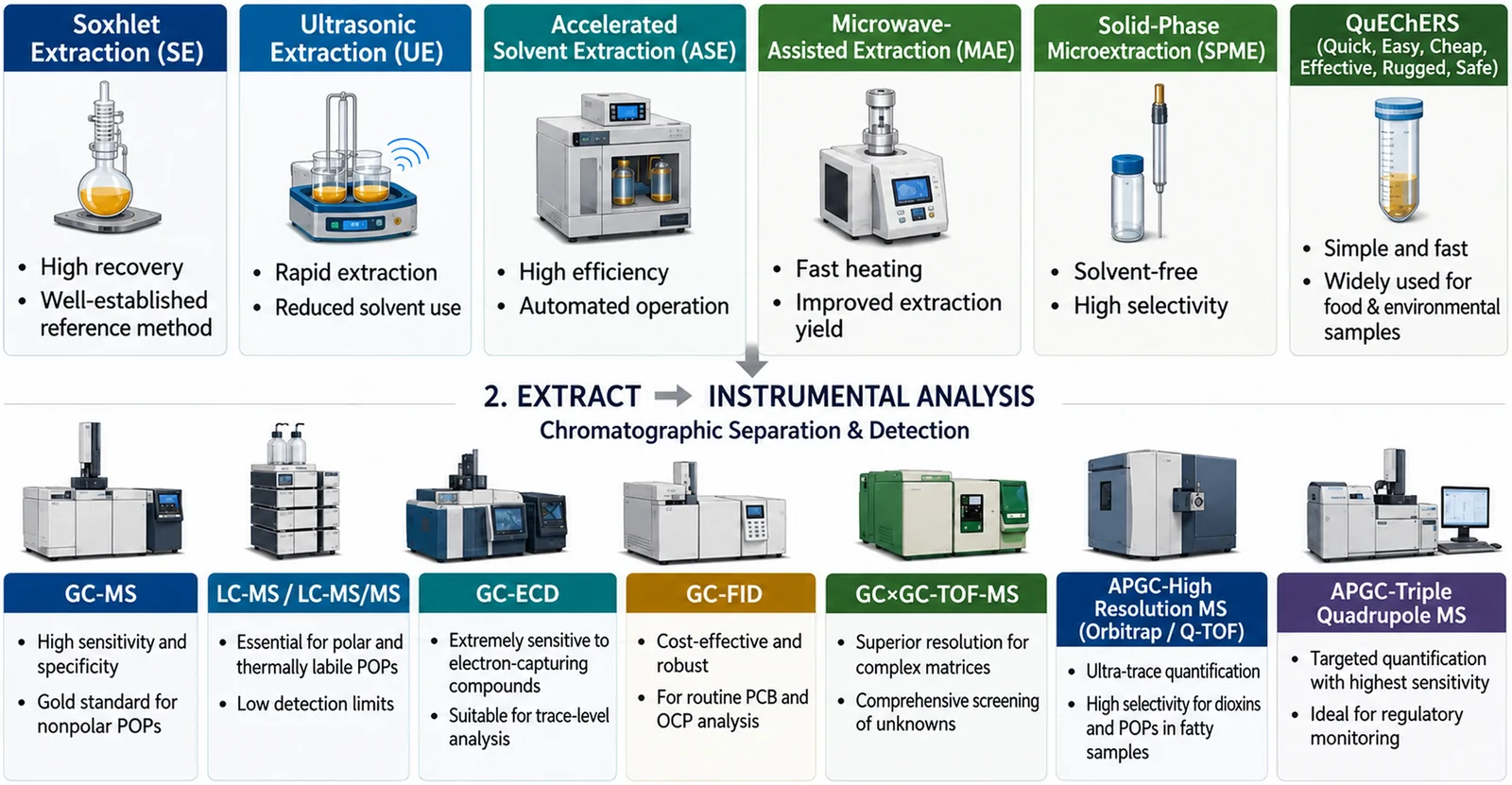
Schematic illustration of analytical workflows for POPs, showing primary extraction techniques and chromatographic detection methods, along with their key analytical advantages

Analyzing POPs in food requires rigorous purification to remove lipids and proteins. Figure 4 illustrates the principal analytical workflows for POPs, integrating extraction techniques with chromatographic separation and detection methods. Techniques such as Quick, Easy, Cheap, Effective, Rugged, and Safe (QuEChERS) and stir bar sorptive extraction (SBSE) are widely used. Advanced methods, such as GC × GC–time-of-flight mass spectrometry (TOF–MS) and atmospheric pressure gas chromatography (APGC)–triple quadrupole MS, offer detection limits comparable to those of HRMS for analytes like dioxins in fatty samples (Focant et al., 2005; Dam et al., 2016; Ábalos et al., 2016). Due to their high polarity, PFAS require analysis by LC–MS/MS (Portolés et al., 2016; Geng et al., 2016). Although GC-HRMS remains the reference method for dioxins, GC × GC–TOF–MS and triple-quadrupole MS/MS are increasingly adopted for their comparable sensitivity and more straightforward operation (L’Homme et al., 2015; Ábalos et al., 2016).

### Biosensors

Biosensors mark a paradigm shift in environmental analysis, providing rapid, on-site detection that offers a compelling alternative to traditional, laboratory-bound chromatographic techniques such as GC–MS and LC–MS. By integrating a biological recognition element (e.g., enzymes, antibodies, whole cells) with a physicochemical transducer, these devices convert specific binding events into quantifiable optical, electrochemical, or thermal signals, thereby potentially reducing  the need for complex sample preparation (Çimen et al., 2023; Naresh & Lee, 2021).

#### Diversity in recognition elements and transduction mechanisms

The versatility of biosensors is evident in the range of biological elements and transduction methods employed. Immunosensors, which leverage the high specificity of antibody–antigen interactions, have been successfully developed for several target analytes. For instance, a piezoelectric immunosensor based on the Quartz Crystal Microbalance (QCM) technique was used to detect various PCB congeners, with a particular emphasis on 4,4′-dichlorobiphenyl (DCB) and structurally related compounds (Přibyl et al., 2006). The recognition elements are polyclonal antibodies immobilized on gold surfaces via oriented attachment through protein A, ensuring specific recognition of PCBs. The transduction mechanism relies on measuring mass-induced frequency shifts in the quartz crystal’s oscillation, which occur as PCBs or their conjugates bind to the immobilized antibodies. The limit of detection ranges from 6 ppb to 1 ppm in both aqueous and organic phases. The system exhibits a rapid response time, typically less than 20 min in aqueous solutions and around 30 min when analyzing samples in organic solvents such as toluene (Přibyl et al., 2006). Stability assessments suggest that the sensors can be reused multiple times with maintained sensitivity; however, an initial antibody loss of about 40% was observed, which stabilized after pre-exposure to toluene. Validation with real-world samples, including soil extracts, demonstrated the biosensor’s practical applicability, with results correlating well with those from high-performance liquid chromatography (HPLC) measurements, although some matrix effects were observed (Přibyl et al., 2006).

In another study, a surface plasmon resonance (SPR) immunosensor was used to detect naphthalene, a representative PAH, and its related naphthol derivatives (Cennamo et al., 2019). In this system, specific antibodies serve as the biological recognition elements, enabling selective binding to naphthalene. The biosensor achieves a detection limit of 0.76 ppb in seawater samples, demonstrating high sensitivity for marine environmental monitoring. The transduction mechanism relies on antibody–antigen interactions, which induce measurable shifts in the SPR resonance wavelength upon analyte binding. These optical changes are directly correlated with naphthalene concentration. Validation with real environmental samples highlights the applicability of this SPR-based immunosensor for monitoring phenolic compounds and PAHs in marine environments (Cennamo et al., 2019).

A whole-cell biosensor employing *Sphingobium xenophagum* has been developed for the selective detection of PBDEs using a bioluminescent transduction mechanism (Chen et al., 2022). This system achieves a detection limit of approximately 10 nM, highlighting its sensitivity and suitability for monitoring PBDE contamination. Using *S. xenophagum* as the biological recognition element represents a novel, target-specific sensing strategy tailored to PBDEs. *S. xenophagum* serves as the recognition element in these biosensors because of its inherent ability to respond selectively to PBDEs. The transduction mechanism is based on bioluminescence, whereby the bacterial cells either naturally or through genetic modification emit light upon exposure to PBDE molecules. Interaction with PBDEs induces specific metabolic or enzymatic responses within the cells, activating the bioluminescent pathway. The resulting light intensity correlates with the PBDE concentration in the sample, enabling quantitative detection (Chen et al., 2022).

An enzyme-based biosensor has been reported for PCB detection, employing peroxidase enzymes immobilized on a polyaniline-modified platinum electrode (Nomngongo et al., 2012). This biosensor demonstrates high analytical sensitivity, with a detection limit of 0.016–0.019 μg/L. The investigation indicated its suitability for monitoring PCBs in landfill leachates and other environmentally relevant samples. Peroxidase or other oxidoreductase enzymes serve as the biological recognition elements and are immobilized on the electrode surface to enable stable, efficient analyte interaction. The transduction mechanism is electrochemical, commonly based on amperometric signal measurement. The enzymatic oxidation of PCB molecules generates changes in current or electrode potential that are proportional to PCB concentration. These PCB-induced redox reactions enable reliable, sensitive detection of PCBs at trace levels (Nomngongo et al., 2012).

A highly selective and reusable whole-cell optical biosensor (WCB) was developed for the semi-quantitative detection of PCBs, specifically those with three chlorine substitutions, and the commercial mixture Delor 103 (D103) (Gavlasova et al., 2008). The biosensor uses *Pseudomonas* sp. P2 cells as the recognition element, immobilized in a silica matrix containing biphenyl. These cells metabolize the target PCBs, including 2,3,4′-trichlorobiphenyl, 2,4,4′-trichlorobiphenyl, and 2,5,4′-trichlorobiphenyl, via their biphenyl catabolic pathway. The transduction mechanism is based on optical detection, monitoring the formation of a distinct yellow meta ring-fission product, 2-hydroxy-6-*oxo*−6-phenylhexa-2,4-dienoic acid (HOPDA), which has a maximum absorbance at 398 nm. The biosensor demonstrated detection limits of 0.5 mg/L for D103 and 0.2 mg/L for the 2,4,4'-trichlorobiphenyl congener (Gavlasova et al., 2008). Detection typically requires a 3-h incubation. The WCB exhibited reusability for up to four uses and maintained storage stability for 2 weeks at 10 °C. Real-sample validation included the analysis of mineral oil and landfill soil samples, with results compared to those obtained by gas chromatography with electron capture detection (GC-ECD) (Gavlasova et al., 2008).

Genetically engineered *Pseudomonas fluorescens* F113 derivative strains, specifically F113rifPCBgfp and F113L::1180gfp, were designed as innovative whole-cell biosensors to detect PCB bioavailability and biodegradation (Power et al., 2011). These biosensors are engineered to detect key PCB degradation intermediates, such as 3-chlorobenzoate (3-CBA) and 3-chlorobiphenyl (3-CBP). The detection mechanism relies on the induction of green fluorescent protein (GFP) expression. When the target analytes are present, they activate the gfp gene, resulting in fluorescence from the bacterial cells. This fluorescence can be quantified, with intensity directly corresponding to the concentration of the inducing compound. These biosensors demonstrated 100% recovery of viable cells even after 250 days of storage under non-sterile, ambient conditions. The study shows that these biosensors can be used to assess biodegradation potential in PCB-contaminated soils (Power et al., 2011).

A genetically engineered bacterial biosensor was designed to detect perfluorooctanoic acid (PFOA) and perfluorooctanesulfonic acid (PFOS) (Sunantha & Vasudevan, 2021). The recognition mechanism depends on integrating a defluorinase gene, which serves as the regulatory element. The defluorinase enzyme is specifically activated by fluorinated compounds, such as PFOA and PFOS. Upon activation of the defluorinase gene by the target POPs, a green fluorescent protein gene (gfp) is expressed. The resulting green fluorescent protein then emits a measurable signal that can be quantitatively assessed with a spectrofluorometer. This enables sensitive detection, with a limit of detection of 10 ng/L and a response achievable up to 1000 ng/L. The biosensor requires 24 h to reach its maximum fluorescence intensity. Its effectiveness has been validated in real environmental water samples, including river water, wastewater, and drinking water (Sunantha & Vasudevan, 2021).

**Fig. 5:**
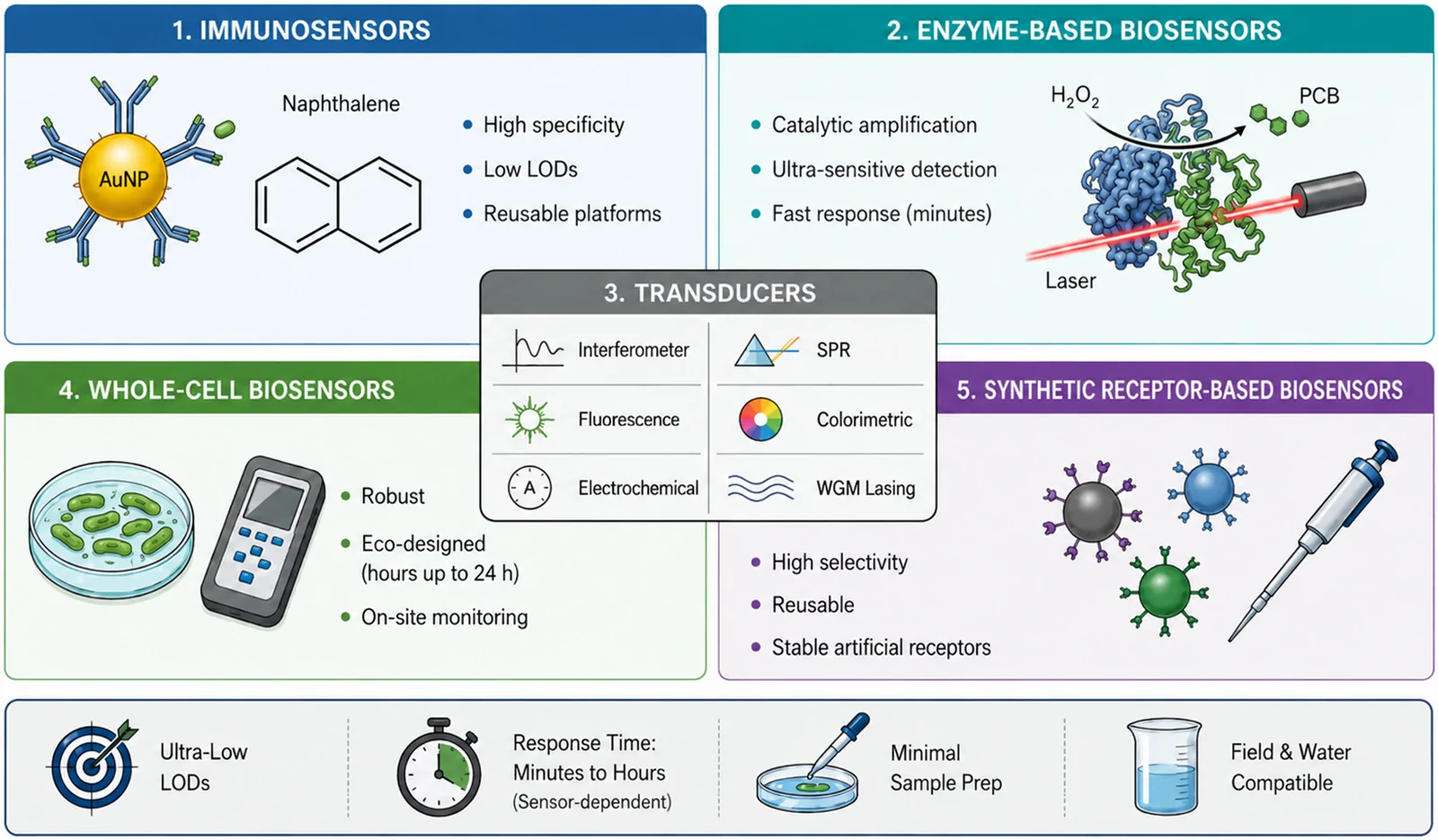
Schematic overview of major biosensing strategies for detecting and monitoring POPs

A comparative summary of the performance characteristics of the discussed biosensors is presented in Table 1, and an integrative summary of the biosensor classes and their corresponding transduction principles is shown in Fig. 5.

**Table 1:** Quantitative performance comparison of biosensor technologies for POP detection

Sensor type	Target analyte	Limit of detection (LOD)	Response time	Stability	Real sample validation	Ref
Piezoelectric immunosensor	PCB congeners (especially 4,4′-dichlorobiphenyl and related congeners)	6 ppb–1 ppm (aqueous and organic phases)	< 20 min (aqueous); ~ 30 min (organic phase)	Reusable; initial ~ 40% antibody loss stabilized after toluene pre-exposure	Soil	(Přibyl et al., 2006)
SPR immunosensor	Naphthalene and naphthol derivatives	0.76 ppb (seawater)	Not mentioned	Stable over several days	Seawater and marine samples	(Cennamo et al., 2019)
Whole-cell bioluminescent biosensor (*Sphingobium xenophagum*)	PBDEs	~ 10 nM	Not mentioned	Not mentioned	Not mentioned	(Chen et al., 2022)
Enzyme-based electrochemical biosensor (peroxidase on polyaniline-modified Pt electrode)	PCBs	0.016–0.019 μg/L	Not mentioned	Not mentioned	Landfill leachates and environmental samples	(Nomngongo et al., 2012)
Whole-cell optical biosensor (*Pseudomonas* sp. P2 immobilized in silica matrix)	Trichlorinated PCBs (e.g., 2,3,4′-, 2,4,4′-, 2,5,4′-CBP) and Delor 103	0.5 mg/L (D103); 0.2 mg/L (2,4,4′-CBP)	~ 3h	Reusable up to 4 cycles; stable for 2 weeks at 10 °C	Mineral oil and landfill soil	(Gavlasova et al., 2008)
Genetically engineered whole-cell biosensor (*Pseudomonas fluorescens* F113 derivatives)	PCB degradation intermediates (3-CBA, 3-CBP)	Not mentioned	Not mentioned	100% viable cell recovery after 250 days	Soil	(Power et al., 2011)
Genetically engineered bacterial fluorescent biosensor	PFOA and PFOS	10 ng/L	~ 24h	Not mentioned	River water, wastewater, and drinking water	(Sunantha & Vasudevan, 2021)

#### Advancements in transducer technology and sensor design

Beyond the established biosensor platforms discussed above, aptamer-based sensors and molecularly imprinted polymer (MIP) sensors have emerged as promising alternatives for POP detection. Aptamers are single-stranded deoxyribonucleic acid (DNA) or ribonucleic acid (RNA) oligonucleotides that bind target analytes with high affinity and specificity comparable to antibodies, while offering advantages such as ease of chemical synthesis, thermal stability, and reversible denaturation (Kerler, 2025). MIPs are synthetic polymers with tailored recognition cavities complementary to target molecules in size, shape, and functional groups, providing robustness, low cost, and resistance to harsh environmental conditions (Spivak, 2010). Both technologies address key limitations of conventional biorecognition elements, namely, antibody instability and enzyme denaturation, making them particularly attractive for field-deployable environmental monitoring applications, although each presents distinct challenges regarding selectivity, sensitivity, and large-scale commercialization (Naseri et al., 2020). Significant progress has been made in transducer technology, enhancing sensitivity, portability, and practicality. Optical transducers are particularly prominent. These include:

1. Interferometric and plasmonic platforms: The bimodal waveguide (BiMW) interferometer used by Ramirez-Priego et al. (2021) and the SPR plastic optical fiber (POF) sensor developed by Cennamo et al. (2019) enable highly sensitive, label-free detection.
2. Fluorescence-based systems: Evanescent wave fluorescence (Liu et al., 2019) and fiber-optic sensors with replaceable MINs@PEGDA detectors (Huang et al., 2021) offer high sensitivity and the potential for multi-analyte detection.
3. Colorimetric and reflectance methods: Simpler optical setups, such as reflectance spectrophotometry, used by Shahar et al. (2019) and direct color change monitoring in algal biosensors (Gosset et al., 2019; Scognamiglio et al., 2019), enable cost-effective, field-deployable solutions.
4. Advanced lasing systems: Whispering gallery mode (WGM) lasing from liquid crystal microdroplets, developed by Duan et al. (2020), represents a cutting-edge approach that pushes the boundaries of detection sensitivity.

A critical trend across these studies is the emphasis on practical applicability. Researchers consistently validated their biosensors with real-world water samples (tap, river, and seawater) and demonstrated performance comparable to standard methods, such as GC-ECD or recombinant yeast bioassays. Key practical advantages frequently reported include rapid analysis (e.g., 20 min for fenitrothion), minimal sample pre-treatment, and excellent recovery rates (e.g., 96 ± 5% for nanoatrazine). Furthermore, integrating biosensors into microfluidic assemblies and developing reusable or stable, single-use platforms underscores their potential for automated, on-site, and continuous environmental monitoring.

Despite their demonstrated analytical potential, biosensors for detecting POPs still face significant practical limitations that hinder large-scale, long-term deployment. A major bottleneck remains the stability of biological recognition elements. Although nanomaterials such as gold nanoparticles have been used to enhance immobilization and signal stability (Naresh & Lee, 2021), many biosensors still rely on enzymes, antibodies, or living cells that are inherently sensitive to environmental stress. Enzymes may denature, antibodies can degrade gradually, and whole-cell systems may lose metabolic activity over time, ultimately compromising sensor reliability and operational lifespan under field conditions (Huang et al., 2023; Patial et al., 2025). Even in well-designed immunosensors, such as QCM- and SPR-based platforms for PCB and PAH detection (Přibyl et al., 2006; Cennamo et al., 2019), partial loss of antibody activity and matrix-induced effects have been observed during reuse or solvent exposure, underscoring the need for more robust recognition strategies.

Matrix interference is another major challenge. Real environmental and biological samples contain complex mixtures of organic matter, salts, particulates, and competing compounds that can cause non-specific binding, surface fouling, or signal suppression. These effects often reduce specificity and accuracy when biosensors are deployed outside controlled laboratory conditions (Huang et al., 2023; Farzin et al., 2025). Although several studies have successfully validated biosensors using real matrices such as seawater, landfill leachates, soil extracts, and wastewater (Přibyl et al., 2006; Cennamo et al., 2019; Nomngongo et al., 2012; Gavlasova et al., 2008; Power et al., 2011; Sunantha & Vasudevan, 2021), matrix effects remain a persistent limitation, underscoring the need for improved surface chemistries, antifouling materials, and standardized validation protocols.

A further limitation stems from trade-offs among performance metrics across biosensor classes. Immunosensors and enzyme-based biosensors generally offer rapid response times (minutes) and high sensitivity, as exemplified by electrochemical PCB sensors with detection limits in the nanogram-per-liter range (Nomngongo et al., 2012) and optical SPR platforms capable of ppb-level detection (Cennamo et al., 2019). In contrast, whole-cell biosensors using bacteria or algae provide ecological relevance by responding to bioavailable fractions of POPs, as demonstrated for PBDEs, PCBs, and PFAS compounds (Chen et al., 2022; Gavlasova et al., 2008; Power et al., 2011; Sunantha & Vasudevan, 2021). However, these systems typically exhibit longer response times, ranging from several hours to 24 h, due to the need for metabolic activation and reporter gene expression. This creates an inherent trade-off between sensitivity and speed or specificity that must be carefully balanced depending on the intended application (Amaral et al., 2024; Chin Chwan Chuong et al., 2022; Xiao et al., 2025).

In addition, a scalability gap persists between laboratory demonstrations and real-world deployment. Many biosensors are validated using spiked or purified samples under short-term testing conditions, whereas long-term monitoring in dynamic environments, characterized by fluctuating pH, temperature, biofouling, and complex interferences, remains limited (Huang et al., 2023; Farzin et al., 2025). Although recent advances in transducer technology and sensor design, including plasmonic, interferometric, fluorescence-based, and microfluidic-integrated platforms (Cennamo et al., 2019; Ramirez-Priego et al., 2021; Liu et al., 2019; Huang et al., 2021; Shahar et al., 2019; Duan et al., 2020), have improved sensitivity, portability, and ease of use, widespread field adoption still requires rigorous, long-duration validation studies.

Collectively, these limitations underscore the need for next-generation biosensors that incorporate more robust recognition elements, such as aptamers, MIPs, genetically stabilized enzymes, and engineered whole-cell systems, as well as advanced materials, including metal–organic frameworks and nanocomposites, engineered for enhanced environmental resilience.

In parallel, AI offers a complementary and transformative advantage for POP monitoring by fundamentally changing how environmental data is acquired, analyzed, and acted upon. Traditional analytical methods, such as GC–MS, remain laboratory bound and time intensive, often requiring extensive sample preparation (e.g., SE, which can last approximately 24 h) followed by lengthy instrumental analysis (Yagishita et al., 2025). Consequently, these approaches provide only discrete temporal snapshots of contamination and fail to capture real-time dynamics or interactions among multiple pollutants (Popescu et al., 2024; Jovanović et al., 2025).

By contrast, AI-enabled monitoring frameworks can integrate data streams from biosensors and other analytical platforms to enable real-time, continuous surveillance and advanced data modeling (Rabbi, 2025). Recent AI-powered screening approaches have delivered on-site results in about 10 min, representing a substantial reduction in analytical turnaround time compared with conventional workflows (Yagishita et al., 2025). Moreover, AI excels at extracting patterns from complex, multivariate datasets, enabling holistic analysis of pollutant mixtures, exposure pathways, and biological interactions that extend beyond the quantification of single compounds (Jovanović et al., 2025; Rabbi, 2025).

Beyond enhanced sensing, AI fundamentally shifts environmental monitoring from a retrospective, descriptive paradigm to a predictive, proactive management framework. ML algorithms can forecast future pollution trends using environmental, industrial, and meteorological variables (Alotaibi & Nassif, 2024), identify pollution sources for targeted mitigation (Popescu et al., 2024), and perform real-time anomaly detection to flag unexpected emission events. When integrated into IoT architectures, biosensors serve as distributed sensing nodes, while AI systems serve as the analytical and decision-making backbone, enabling automated alerts, adaptive monitoring strategies, and closed-loop remediation responses (Popescu et al., 2024; Alotaibi & Nassif, 2024).

The transformative advantages of AI-enabled POP monitoring over conventional laboratory-based approaches are summarized in Table 2, highlighting improvements in response time, spatial and temporal resolution, predictive capability, and operational integration. Together, advances in biosensor technology and AI-driven analytics represent a convergent pathway toward intelligent, resilient, and scalable environmental monitoring systems.

**Table 2:** Comparative analysis of conventional and AI-enabled systems for POP monitoring

Monitoring metric	Conventional methods (GC–MS/LC–MS)	AI-enabled systems	Ref
Detection limit	GC–MS/LC–MS (with HRMS) achieves extremely low detection limits (fg–pg levels) for POPs, such as dioxins, PCBs, PBDEs, and PFAS, in environmental/biological matrices (air, water, soil, biota). High-resolution GC-HRMS and LC-HRMS enhance sensitivity and selectivity	AI/ML-assisted spectral analysis and sensor modeling can improve the limit of detection by refining signal classification, reducing noise, and enabling pattern recognition in complex field data. Emerging AI-biosensor systems show promise for higher apparent sensitivity and rapid identification	(Popescu et al., 2024; Thanigaivel et al., 2025; Avino & Russo, 2018)
Sample throughput and preparation time	Traditional sample prep (SPE, Soxhlet) and offline chromatography are time consuming. Chromatography runs per sample can be multi-minute with extensive clean-up	Sensor networks with AI/IoT can acquire continuous multi-parameter data streams and automate feature extraction; potential for rapid, autonomous field sampling	(Popescu et al., 2024)
Temporal resolution	Discrete sampling campaigns yield periodic snapshots (e.g., quarterly GMP air samplers)	AI-enabled sensor networks could allow near real-time monitoring and anomaly detection across sensor arrays	(Popescu et al., 2024; Fiedler et al., 2024)
Analytical latency	Days to weeks (collect → transport → prep → analysis)	Seconds to hours (edge/AI processing, continuous sensors)	(Alotaibi & Nassif, 2024)
Operational model	Centralized laboratories, highly skilled personnel	Decentralized, in-field deployment with autonomous edge processing	(Popescu et al., 2024)
Data utilization	Primarily descriptive/trend analysis for reporting and regulatory compliance	Predictive/diagnostic analytics: anomaly detection, temporal forecasting, source attribution from multivariate sensor data	(Alotaibi & Nassif, 2024)

A comparative summary of the performance characteristics of the biosensor classes discussed is presented in Table 3, which systematically contrasts their recognition elements, advantages, limitations, and typical applications. Collectively, these biosensor platforms present distinct trade-offs that must be carefully evaluated for specific monitoring applications. Immunosensors and enzyme-based biosensors, the most technologically mature classes, provide exceptional specificity and sensitivity (Zhang et al., 2026; Morawska et al., 2025) but are constrained by the inherent instability of biological recognition elements, limiting their operational lifespan under field conditions (Zhang et al., 2026; Morawska et al., 2025). Aptamers and MIPs offer promising alternatives, combining high selectivity with significantly enhanced stability, reusability, and resistance to environmental stressors, making them increasingly attractive for long-term field deployment (Shang et al., 2026; Seo et al., 2025). However, aptamer screening remains resource intensive (Yang et al., 2026), while MIPs may suffer from template leakage and non-specific binding in complex environmental matrices (Patra et al., 2024). Whole-cell biosensors uniquely capture bioavailability and toxicological effects, yet their prolonged response times and complex biological maintenance requirements severely constrain real-time monitoring applications (Xi et al., 2025). Therefore, sensor selection must be guided by the specific monitoring objectives: rapid sensitivity and specificity (favoring immunosensors/aptamers), long-term stability in harsh conditions (favoring MIPs), or ecotoxicological assessment (favoring whole-cell systems) (Zhang et al., 2025). Hybrid approaches that combine multiple recognition elements or transduction mechanisms may offer synergistic advantages for comprehensive POP monitoring, though such integration introduces additional complexity and validation requirements (Cetinkaya et al., 2026; Yang et al., 2026).

**Table 3:** Comparative analysis of biosensor technologies for POP detection

Sensor type	Recognition element	Key advantages	Key limitations	Typical applications	Ref
Immunosensors	Antibodies	High specificity and affinity; well-established clinical validation	High cost; environmental sensitivity (pH, temperature); limited reusability; matrix interference	Confirmatory analysis requiring high specificity	(Torrinha et al., 2024)
Enzyme-based sensors	Enzymes (e.g., peroxidase, laccase, acetylcholinesterase)	High specificity; catalytic signal amplification; rapid response	Limited stability; pH/temperature sensitivity; short shelf life; denaturation risks	Detection via specific enzymatic reactions	(Zhang et al., 2026)
Aptamer sensors	DNA/RNA aptamers	High specificity/affinity; facile chemical synthesis; thermal stability; reversible denaturation; reusability	SELEX screening is expensive and time consuming; performance may decrease in complex matrices	Applications requiring high specificity and stability	Naseri et al., 2020; Li et al., 2026)
MIP sensors	Synthetic polymer with tailored cavities	High robustness; low cost; excellent chemical stability; resistance to harsh conditions	Incomplete template removal possible; potential non-specific binding; limited selectivity for structurally similar compounds	Harsh environmental conditions (water, soil); field-deployable monitoring	Naseri et al., 2020; Liu et al., 2026)
Whole-cell biosensors	Living microorganisms (bacteria, algae)	Provides bioavailability and eco-toxicological information; detects complex mixture effects; self-regenerating	Long response times; complex cell maintenance; variable specificity; limited field stability	Ecological risk assessment; bioavailability monitoring	Cheliukanov et al., 2025)

### Advanced monitoring and sensing technologies

#### Sensor networks and IoT

The IoT is conceptualized as a transformative framework for intelligent environmental protection. By leveraging communication technologies and distributed sensors, the IoT creates a sophisticated network that interconnects devices and systems, enabling the remote management and intelligent operation of environmental monitoring platforms. A pivotal application is the Great Lakes Integrated Atmospheric Deposition Network (IADN), which uses an IoT-based infrastructure to systematically collect long-term, high-resolution data on atmospheric pollutants. This capability is fundamental to data-driven research, providing the extensive and reliable datasets required for advanced analysis. Furthermore, the IoT ecosystem facilitates the entire research workflow, from the initial collection of environmental data and its acquisition from online repositories to the application of ML for analysis and the subsequent sharing of results, underscoring its pivotal role in making environmental management more scientific, efficient, and digitized (Wu et al., 2021).

Building on this foundational potential, recent research demonstrates the advanced analytics enabled by IoT data. Yu et al. (2022) proposed an intelligent system for predicting POP concentrations by integrating the Autoregressive Integrated Moving Average (ARIMA) model with Long Short-Term Memory (LSTM) networks. This hybrid approach was designed to model both linear and nonlinear components of the POP time series. Their methodology included preprocessing with a one-class support vector machine (SVM) algorithm for outlier detection and parameter optimization via the Bayesian Information Criterion for ARIMA, as well as a grid search for LSTM. Validated using IADN data from the Great Lakes region, the combined ARIMA–LSTM model achieved superior predictive accuracy, as evidenced by lower mean absolute error (MAE), root mean squared error (RMSE), and symmetric mean absolute percentage error (SMAPE) values than standalone models. The system also forecast a general decline in POP concentrations over the next 5 years, with seasonal fluctuations, highlighting the potential of hybrid models in IoT-enabled environmental forecasting (Yu et al., 2022).

Complementing predictive analytics, IoT also enhances real-time chemical detection. Jeong and Choi focused on AI-based toxicity prediction for environmental chemicals, with significant implications for POP monitoring. Their work emphasized electronic nose (E-nose) technologies, which use sensor arrays to generate a unique “smellprint” when volatile compounds are encountered. The core innovation applies advanced pattern recognition, ANNs, and fuzzy logic to deconstruct these complex signals and identify specific pollutants. This AI-driven approach enables real-time detection and discrimination of hazardous substances directly in the field, offering a significant advantage over traditional, time-consuming laboratory methods for applications such as urban air quality monitoring and industrial leak detection (Jeong & Choi, 2022).

The practical deployment of these concepts depends on a robust network architecture. Mutunga et al. found that integrating Sensor Networks and IoT is pivotal for real-time, large-scale monitoring of pesticides in water. Their key finding was that technologies like LoRa enable long-range data transmission from sensor nodes over distances of up to 15 km. They concluded that wireless sensor networks (WSNs) provide a scalable platform for vast areas, while IoT architectures facilitate seamless data flow to the cloud using lightweight protocols such as Message Queuing Telemetry Transport (MQTT) and Constrained Application Protocol (CoAP). However, they also highlighted critical challenges, including a scarcity of commercially available pesticide sensors, the need for greater energy efficiency, and the imperative for data security measures (Mutunga et al., 2024).

Finally, Zhao and Jiang demonstrated a complete IoT system that integrates sensing, analysis, and response. They developed a comprehensive monitoring and management system for POPs in subway station groundwater. The method used a sensor network measuring water quality parameters and specialized sensors for chlorophenol compounds. An IoT-based early warning module sent automatic alerts when pollutant levels exceeded thresholds and was integrated with governance equipment, such as a photocatalytic ozone flow reactor (PCOFR), for automated treatment. The results were highly effective, with the PCOFR achieving degradation efficiencies of 93% for 2-chlorophenol, 86% for 2,4-dichlorophenol, and 83% for 2,4,6-trichlorophenol. The system also significantly reduced heavy metal ion concentrations and other pollutants, confirming its utility for real-time monitoring and active water quality improvement (Zhao & Jiang, 2024).

Collectively, these studies illustrate a robust and evolving paradigm for environmental monitoring. The convergence of sensor networks and the IoT provides the foundational infrastructure for collecting high-resolution, real-time data across vast geographic scales, as seen in networks like the IADN. This data-rich environment enables the application of sophisticated AI and hybrid statistical models, moving beyond simple data collection to accurate forecasting of pollutant trends, as demonstrated by Yu et al. (2022). Furthermore, integrating AI with specialized sensor hardware, such as E-noses, enables immediate, on-site hazard identification, transforming monitoring from a delayed analytical process into an instantaneous diagnostic tool. The research by Zhao and Jiang (2024) represents the ultimate expression of this paradigm: a closed-loop system in which the IoT not only detects and analyzes pollution but also automatically triggers and manages remediation efforts. As noted by Mutunga et al. (2024), fully achieving this potential depends on addressing several significant technical challenges. These include the need for more reliable and diverse chemical sensors, improved energy self-sufficiency for remote monitoring units, and stronger cybersecurity measures. Overall, future developments are expected to move toward more intelligent and more automated environmental management systems, in which data collection, analysis, and decision-making are closely integrated.

Economic considerations reveal shifting cost paradigms between conventional and AI-enabled monitoring. Traditional GC–MS analysis is costly; commercial laboratory pricing ranges from $150 to $800 per sample, with routine analysis often falling between $150 and $300. This includes labor, consumables, and instrument time, and turnaround times are typically measured in days (Sinha, 2026). In contrast, AI-enhanced screening methods are recognized for reducing costs and providing faster results, though often at the expense of quantitative precision, making them complementary to confirmatory techniques like GC–MS.

Life-cycle costs for deploying IoT sensor networks must account for the total cost of ownership (TCO), which includes initial hardware, cellular connectivity, ongoing maintenance, and operational management over the system’s lifetime (Fourie, 2025). A major factor influencing these costs is sensor reliability. Biofouling is widely recognized as the primary factor degrading sensor performance, maintenance, and data quality in aquatic environments, with estimates that up to 50% of operational budgets can be attributed to addressing fouling-related issues. This necessitates design considerations such as redundancy and frequent maintenance, which increase both capital and operational expenditures (Delgado et al., 2021). Therefore, a break-even analysis comparing networked sensors to traditional grab sampling must be based on a comprehensive TCO model for the specific application, considering the required spatial coverage and temporal frequency (Fourie, 2025).

**Fig. 6:**
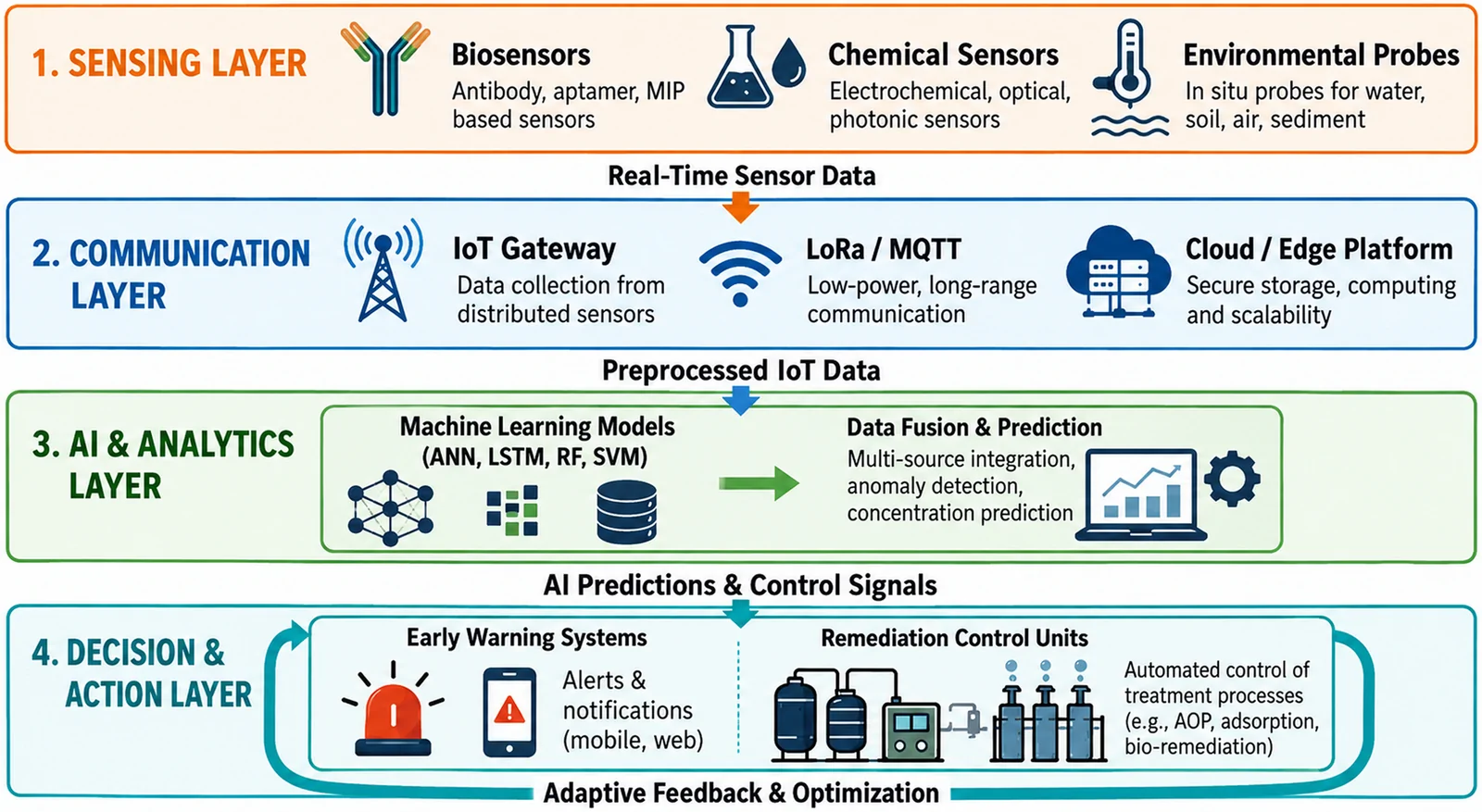
AI–IoT system architecture illustrates the data flow from environmental sensing hardware to ML analytics, decision support, and remediation control

Figure 6 illustrates the integrated architecture of AI- and IoT-enabled environmental monitoring systems, highlighting the flow of data from distributed sensing hardware to advanced analytics and decision-making modules. The framework emphasizes how real-time sensor data are transformed, through communication and AI layers, into actionable insights and adaptive remediation control.

#### Machine learning and data analysis

The application of ML and advanced data analysis is significantly enhancing environmental monitoring by enabling the prediction and identification of pollutants through complex, nonlinear patterns in data, often reducing reliance on costly and time-consuming laboratory analyses. This paradigm shift enables near-real-time assessment, providing deeper insights into the sources and dynamics of contamination.

Zhang et al. applied Quantitative Structure–Activity Relationship (QSAR) modeling to predict the air half-lives of POPs. Using partial least squares (PLS), multiple linear regression (MLR), and genetic function approximation (GFA) on a set of 60 POPs, they identified five key molecular descriptors. The MLR model performed best, achieving remarkable *R*^2^ values of 0.939 and 0.940 for predicting the mean and maximum half-lives, respectively, providing a robust computational tool for assessing POP persistence without extensive experimentation (Zhang et al., 2023). Similarly, to predict the temporal dynamics of POPs in the environment, Wu et al. developed an advanced framework to forecast concentrations in the Great Lakes. They evaluated models including ARIMA, linear regression, XGBoost, and LSTM networks. To address limitations of conventional metrics, they introduced a Penalty Dynamic Time Warping (Penalty-DTW) score and integrated deep ensemble techniques for uncertainty quantification. Their enhanced LSTM model significantly outperformed all other methods, achieving the lowest test RMSE (5.16) and Penalty-DTW score (35.39). The model confirmed a declining trend in PCBs and revealed a strong seasonal pattern, with summer and fall concentrations approximately 40% higher than those in winter (Wu et al., 2021).

Moreover, other studies have leveraged ML for source identification and exposure analysis. Ekpe et al. identified 252 organic micropollutants in groundwater from four distinct regions. To trace contamination sources, they combined a bootstrapped feature selection algorithm with a Partial Least Squares Discriminant Analysis (PLS-DA) model. This workflow demonstrated high predictive performance (*Q*^2^ = 0.897) and pinpointed 51 chemical indicators, successfully linking them to sources such as oil, landfills, agriculture, and industry (Ekpe et al., 2024). In a toxicological context, Jovanovic et al. applied an AI framework to analyze POPs in human breast milk. Their optimized ExtraTrees model achieved high accuracy (*R*^2^ = 0.91) in predicting PCB-170 concentrations. Using explainable AI (XAI) techniques, they identified the dominant predictive congeners (PCB-180, PCB-153, PCB-138) and revealed significant interaction effects, advocating a shift to multi-pollutant exposure assessments (Jovanović et al., 2025).

Finally, novel screening methodologies are emerging that integrate analytical instrumentation with AI. Yagishita et al. developed a rapid screening tool for POPs in waste materials by coupling excitation–emission matrix (EEM) fluorescence spectroscopy with a convolutional neural network (CNN), specifically AlexNet. The system classified samples with 75% accuracy and high specificity, detecting POPs at concentrations as low as 1/1250th of the standard limit. The entire process takes approximately 10 min, offering a significant speed advantage over traditional techniques, such as GC/MS, for qualitative on-site screening (Yagishita et al., 2025; Wu et al., 2021).

In summary, the collective findings from these studies highlight the transformative impact of ML on environmental science. The research demonstrates that ML models, including ANNs, SVMs, LSTMs, and ensemble methods, can achieve high accuracy in predicting pollutant concentrations and persistence using correlated, easier-to-measure parameters. Furthermore, ML techniques are proving invaluable for identifying contamination sources, deciphering complex exposure patterns, and enabling rapid, novel screening technologies. While not a replacement for precise quantitative analysis, these approaches provide efficient, cost-effective, and insightful tools for enhancing environmental monitoring, informing regulatory strategies, and ultimately protecting ecosystem and human health.

ML applications in POP monitoring reveal important methodological constraints. Data scarcity fundamentally limits model generalizability, a recognized challenge across environmental ML studies (Georgiades et al., 2025). Spatial transferability is another critical gap; for instance, ML models trained on specific regional data may fail in different environmental regimes due to region-specific transport dynamics. The influence of interfering environmental variables often remains unaddressed. For example, while Ekpe et al. successfully identified groundwater pollution sources by combining suspect/non-target screening with ML (PLS-DA) and achieved good predictive ability (*Q*^2^ of 0.897), their proposed workflow for classifying contamination sources did not explicitly address temporal variables such as seasonal hydrological fluctuations in the published methodology (Ekpe et al., 2024). Contradictory findings regarding optimal model architectures are common, as performance is highly dataset specific. This is illustrated by research on POP concentration prediction in the Great Lakes, where a hybrid ARIMA–LSTM model was developed and found to be superior for capturing both linear and nonlinear components in the time series data (Yu et al., 2022). These limitations underscore the need for larger, geographically diverse training datasets and standardized benchmarking protocols across environmental domains. They also indicate that differences in reported model performance should be interpreted cautiously because apparent superiority may reflect differences in dataset composition, validation design, and environmental context rather than intrinsic differences in algorithmic capability.

**Fig. 7:**
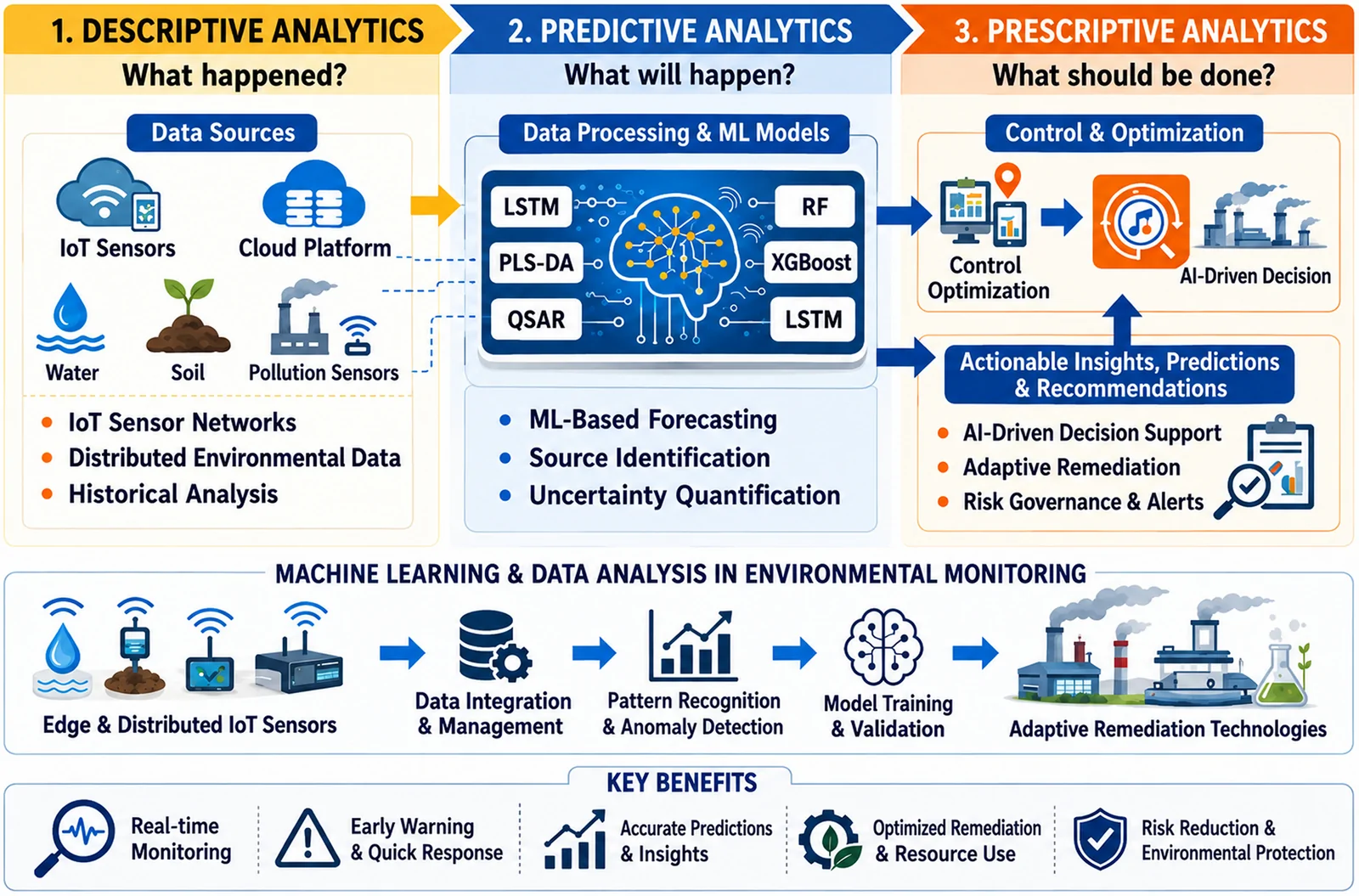
ML-driven transition from descriptive to prescriptive analytics for POP monitoring and management

The studies reviewed demonstrate a clear trend: from descriptive analytics (what happened) to predictive analytics (what will happen) and toward prescriptive analytics (what should be done). This progression from descriptive monitoring to predictive and prescriptive intelligence is schematically illustrated in Fig. 7, highlighting ML’s central role in transforming environmental data into actionable decision support.

#### Comparative framework for ML algorithm selection

The choice of an appropriate ML algorithm for POP monitoring and remediation depends on the characteristics of the available data and the specific management objective. ML approaches have been applied to POP concentration prediction, chemical identification, biodegradation-rate estimation, and contaminant-removal or adsorption prediction (Yu et al., 2022; Cheng et al., 2024; Karbassiyazdi et al., 2022; Mahfouz et al., 2024). Comparative evidence indicates that tree-based models can be highly competitive for medium-sized tabular datasets, whereas the suitability of more complex architectures depends on the structure and characteristics of the available data (Grinsztajn et al., 2022).

For structured POP datasets, Random Forest (RF) and gradient-boosting approaches are important options for modeling nonlinear relationships. In POP prediction, RF demonstrated the highest overall performance among RF, XGBoost, SVM, and neural-network models evaluated, and Shapley Additive Explanations (SHAP) analysis was used to examine predictor contributions to model outputs (Mahfouz et al., 2024). XGBoost has also been applied to contaminant-removal prediction, including predicting PFAS adsorption capacity, equilibrium, and removal performance from material and operating characteristics (Karbassiyazdi et al., 2022). LightGBM provides an additional gradient-boosting approach and has demonstrated strong performance for POP identification using molecular descriptors, with performance better than or comparable to a deep convolutional neural network (DCNN) in the evaluated application (Xin et al., 2024).

SVM/support vector regression (SVR) remains applicable to classification and regression problems and has been evaluated alongside RF, XGBoost, and neural-network approaches for POP prediction (Mahfouz et al., 2024). ANN/multilayer perceptron (MLP) models offer flexible nonlinear modeling but entail greater complexity; therefore, their value relative to simpler approaches should be established through comparative validation rather than assumed from model complexity alone (Karbassiyazdi et al., 2022; Mahfouz et al., 2024).

For temporal POP monitoring, LSTM provides an appropriate framework for modeling sequential information. An ARIMA–LSTM framework has been applied directly to POP concentration prediction in the Great Lakes, combining ARIMA for linear components with LSTM for nonlinear components of the concentration series (Yu et al., 2022). CNN-based approaches are particularly relevant when POP-related information is represented using structured molecular or other high-dimensional representations. A DCNN based on two-dimensional molecular descriptors has been used to identify potential PBT/POP-like chemicals (Sun et al., 2020).

Accordingly, algorithm selection should balance dataset characteristics, the prediction or management objective, model complexity, computational requirements, and interpretability. RF and gradient-boosting methods are particularly relevant to structured POP datasets, whereas LSTM is appropriate for temporal POP concentration prediction and CNN-based approaches for structured molecular representations (Yu et al., 2022; Karbassiyazdi et al., 2022; Mahfouz et al., 2024; Xin et al., 2024; Sun et al., 2020). ML approaches have also shown promise for predicting biodegradation half-lives of organic contaminants, though the optimal model can vary with the environmental matrix and dataset characteristics (Cheng et al., 2024). Across ML applications, algorithmic performance is strongly context dependent rather than universally transferable across POP-related datasets and management objectives. For example, RF achieved the strongest performance among the models evaluated for POP concentration prediction, whereas Gradient Boosting Machine (GBM) performed best for photocatalytic PFOA degradation, and LSTM-based approaches provided superior performance for temporal PCB concentration forecasting. These differences should not be interpreted as evidence that one algorithm is intrinsically superior to the others; rather, they reflect differences in data structure, temporal dependence, predictor dimensionality, environmental matrix, sample size, and modeling objective. Similar variability is evident in biodegradation prediction, where classifying fast versus slow degradation achieved substantially higher performance than quantitatively predicting degradation half-lives, indicating that increasing predictive complexity does not necessarily translate into equivalent quantitative reliability. Collectively, these findings demonstrate that reported model performance metrics cannot be directly compared across studies without considering differences in dataset composition, preprocessing, feature selection, validation strategy, and evaluation metrics. Consequently, the current literature supports application-specific algorithm selection rather than a universal model hierarchy. This also highlights an unresolved methodological challenge: the absence of standardized datasets, cross-validation procedures, external validation datasets, and common performance metrics limits reproducible benchmarking and makes it difficult to determine whether reported improvements represent genuine methodological advances or are primarily attributable to dataset-specific characteristics. The comparative characteristics, strengths, limitations, data requirements, interpretability, and representative applications of the principal algorithms are summarized in Table 4, which further illustrates the methodological trade-offs underlying algorithm selection across different POP-related applications.

**Table 4:** Comparative analysis of ML algorithms for POP management applications

Algorithm	Best suited for POP context	Key strengths	Key limitations	Relative data requirement	Interpretability	Ref
RF	Structured POP prediction and classification	• Models nonlinear relationships • Strong comparative performance in POP prediction • Provides variable-importance measures	• Does not inherently model sequential dependence • Performance depends on dataset characteristics and model configuration	Low-medium	Moderate-high	(Mahfouz et al., 2024)
XGBoost	Structured prediction; contaminant-removal and adsorption modeling	• Models nonlinear relationships • Captures interactions among material and operating variables • Effective predictive performance	• Requires appropriate parameter configuration • Does not inherently model sequential dependencies	Low-medium	Moderate	(Karbassiyazdi et al., 2022)
LightGBM	POP identification; structured environmental prediction	• Efficient gradient-boosting framework • Effective for structured predictor data	• Performance depends on dataset characteristics • Requires appropriate parameter configuration	Medium	Moderate	(Xin et al., 2024)
ANN/MLP	Nonlinear POP prediction and related environmental modeling	• Flexible nonlinear function approximation • Can represent complex predictor–response relationships	• Greater model complexity • Comparatively lower interpretability • Performance depends on training data and model configuration	Medium–high	Low	(Mahfouz et al., 2024)
SVM/SVR	POP classification and regression; structured datasets	• Effective nonlinear classification/regression framework • Applicable to relatively limited datasets	• Requires kernel and parameter selection • Computational requirements can increase with dataset size	Low-medium	Low-moderate	(Mahfouz et al., 2024)
CNN/DCNN	Structured molecular and high-dimensional representations	• Automatic feature extraction from structured representations • Suitable for complex input representations	• Greater model complexity • Comparatively limited interpretability	Medium–high	Low	(Xin et al., 2024; Sun et al., 2020)
LSTM	Temporal POP concentration forecasting	• Models sequential information • Captures temporal dependencies	• Requires suitable temporal data • Greater model complexity • Lower interpretability	Medium–high	Low	(Yu et al., 2022)

### Regulatory governance and international frameworks

The global response to POPs is coordinated under the Stockholm Convention, which provides a framework for monitoring and controlling these POPs.

#### Monitoring and evaluation mechanisms

Article 16 establishes the Convention’s effectiveness evaluation mechanism, conducted every 6 years using scientific, technical, environmental, and economic data. The process draws on findings from POP monitoring reports, national implementation updates under Article 15, and compliance assessments under Article 17. The Effectiveness Evaluation Committee (EEC), established in 2015, oversees these evaluations. The first cycle (2010–2016) defined reference baselines, while the second cycle (2017–2023) focused on tracking trends in newer POPs, including short-chain chlorinated paraffins (SCCPs) and hexabromocyclododecane (HBCDD) (Eighth Meeting of the Conference of the Parties to the Stockholm Convention, 2017; Stockholm Convention National Implementation Plans (NIPs), 2025).

The Global Monitoring Plan (GMP) is central to the Convention’s surveillance strategy, providing standardized protocols for monitoring air, water, and human biomonitoring matrices, such as serum and breast milk, particularly for substances like PFOS and PFOA. Remote air sampling serves as an early indicator of regulatory effectiveness, while human biomonitoring assesses internal exposure levels. Initiated in 2009, water monitoring captures long-term environmental responses to PFAS pollution (Muir & Miaz, 2021). Regional Organization Groups (ROGs) synthesize data into global reports by collaborating with long-standing programs, such as the Arctic Monitoring and Assessment Programme (AMAP) and the Great Lakes monitoring networks. Supplementary media, including snow, sediments, and biota, are also evaluated to understand transport pathways and climate-related effects. Although concentrations of legacy POPs, such as PCBs and DDT, have remained stable, newer contaminants, like PBDEs, show concerning upward trends (Stockholm Convention National Implementation Plans (NIPs), 2025; Holoubek et al., 2008).

#### Implementation and compliance

Parties are required to develop and update National Implementation Plans (NIPs) that outline strategies for eliminating POPs. However, as of 2021, more than 70 countries, mainly low- and middle-income nations, had not revised their NIPs, reflecting persistent capacity gaps (Stockholm Convention National Implementation Plans (NIPs), 2025; From NIPs to implementation: Lessons learned report | UNEP - UN Environment Programme, 2018). To address this, 16 Regional Centres provide technical assistance, technology transfer, and guidance on best available techniques (BAT) and best environmental practices (BEP) (2009; BRS Conventions, 2009). Within the European Union, Regulation No 850/2004 integrates the Stockholm Convention into regional law. Complementing this, the EU Water Framework Directive (2000/60/EC) uses Environmental Quality Standards (EQS) and a Watch List mechanism (Decision 2015/495/EU) to monitor established and emerging pollutants, including PFAS (Sousa et al., 2018).

Data transparency is facilitated by digital platforms such as the GMP Data Warehouse, which consolidates global datasets and leverages cheminformatics tools to harmonize heterogeneous information sources (Dulio et al., 2018; Mark et al., 2016). Consistent reporting protocols are essential for evaluating exemption requests, such as the continued use of PFOS in firefighting foams, and for defining low POP concentration limits in waste materials (Seventh Meeting of the Conference of the Parties to the Stockholm Convention, 2015). Additionally, emerging issues, such as the role of microplastics in POP transport, necessitate updates to Annex D of the Convention to better assess long-range environmental transport (LRET) mechanisms (Jang et al., 2017; 16th Meeting of the Persistent Organic Pollutants Review Committee to the Stockholm Convention (POPRC-16), 2025; Sixteenth meeting of the Persistent Organic Pollutants Review Committee (POPRC.16), 2021).

## Treatment technologies and machine learning for the treatment of POPs

### Treatment technologies

**Fig. 8:**
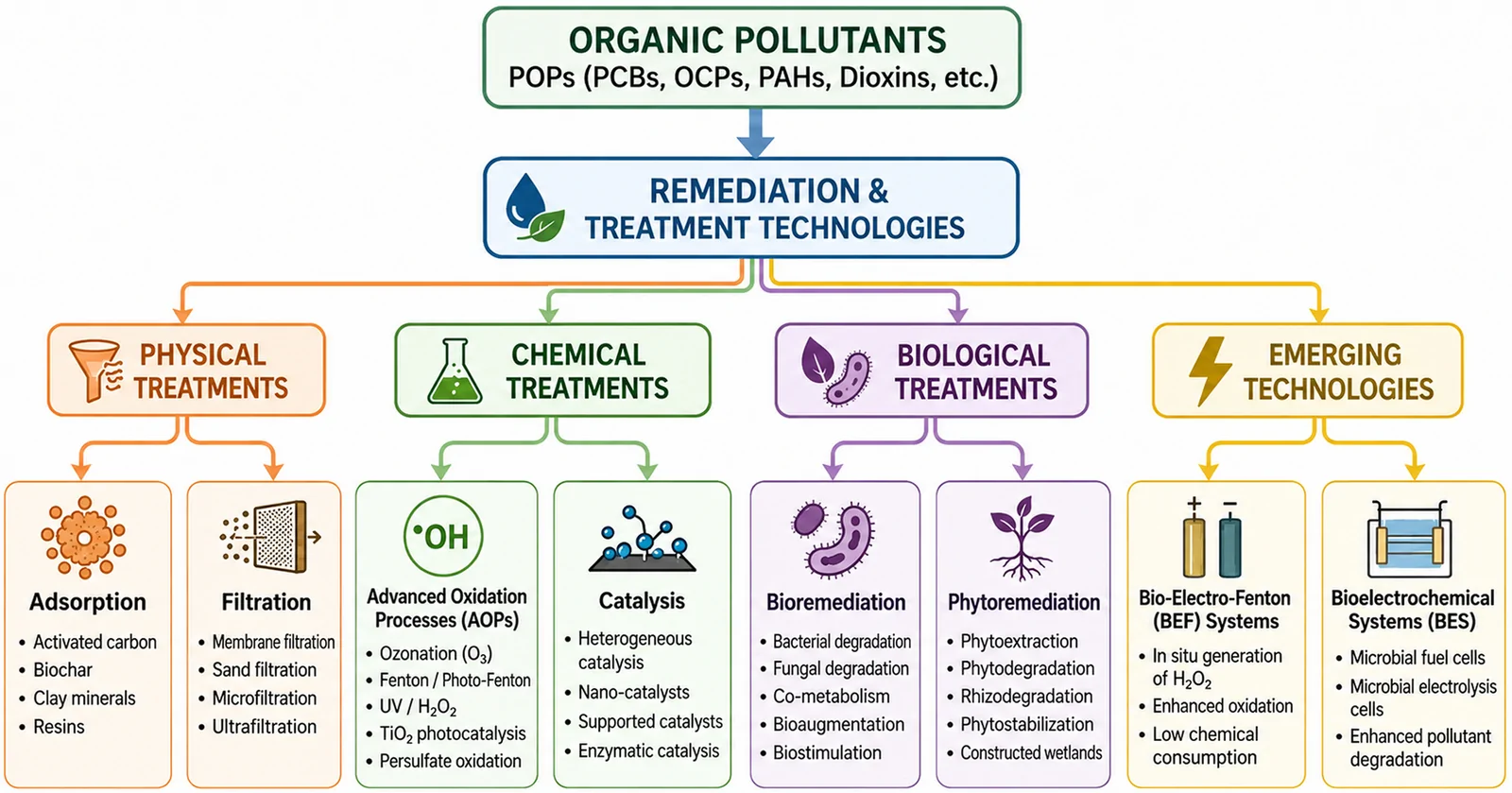
Schematic representation of different water treatment methods

A central challenge in remediating POPs is overcoming their inherent chemical stability. POPs, such as PCBs and dioxins, have strong carbon–halogen bonds that resist natural hydrolysis and photolysis (Paul & Ahmaruzzaman, 2025). Therefore, effective technologies must either break these bonds directly or transform the pollutants into biodegradable forms. Current strategies can be categorized as destructive chemical processes, biological and bio-hybrid systems, physical separation methods, and integrated solutions that combine these approaches for synergistic effects (Fig. 8).

#### Advanced oxidation processes

AOPs generate powerful, non-selective radicals, such as hydroxyl (•OH) and sulfate radicals (SO₄•⁻), capable of oxidizing even the most recalcitrant POPs (Paul & Ahmaruzzaman, 2025). Persulfate-based AOPs are particularly effective, generating a broader spectrum of reactive oxygen species and operating over a wider pH range than traditional Fenton processes. For example, a catalytic system using a MnO_x_-CeO_x_-Al_2_O_3_ catalyst to activate persulfate achieved over 85% removal of diverse POPs from complex landfill leachate by leveraging both radical and non-radical electron transfer pathways (Bai et al., 2026).

Photocatalysis, a subset of AOPs, uses light-activated semiconductors such as TiO_2_ or ZnO to generate oxidizing radicals. Research focuses on enhancing these materials for visible-light activity through doping or by creating heterojunctions (Paul & Ahmaruzzaman, 2025). While highly effective, a key limitation of all AOPs is the potential formation of incomplete oxidation by-products, which may require additional treatment steps (Bai et al., 2026).

#### Biological and bio-hybrid systems

Biological methods leverage microorganisms and plants for sustainable degradation, though their effectiveness against pure POPs often benefits from integration with other technologies. Engineered microbial consortia represent a significant advancement. Combining immobilized, genetically engineered microbes (imGEMs) with catalytic oxidation has shown exceptional results. One system achieved over 96.8% removal of POPs from landfill leachate by first using catalytic persulfate to break down complex molecules, followed by specialized microbes engineered with catabolic genes (e.g., for aromatic ring attack) to mineralize the intermediates. This approach also significantly reduced operational costs and greenhouse gas emissions (Bai et al., 2026).

Microalgae–bacteria consortia offer a synergistic, nature-inspired system for degrading POPs such as PCBs and PAHs. Microalgae produce oxygen through photosynthesis, supporting aerobic bacteria that degrade the pollutants, while the bacteria provide CO_2_ and metabolites for algal growth. This symbiosis yields a more stable and resilient system than single-species approaches (Guo et al., 2024).

Phytoremediation and Circular Bio-Management use plants to sequester POPs from soil and water. A groundbreaking study demonstrated a circular economy model for the resulting contaminated biomass. Fermenting contaminated reed and nettle tissue from constructed wetlands with a specialized microbial consortium removed 87–95% of the sequestered POPs and transformed the waste into a pathogen-resistant liquid fertilizer, thereby closing the waste loop (Butcher et al., 2025).

#### Physical and nanomaterial-based separation

These technologies aim to concentrate or immobilize POPs, often as a first step before their destruction. Adsorption remains a widely used first-step technology due to its simplicity. Advanced nanoadsorbents such as carbon nanotubes (CNTs), graphene oxide, and metal–organic frameworks (MOFs) offer extremely high surface areas and tunable pores for capturing specific POPs. While nanoadsorbents can achieve high removal capacities, they typically concentrate rather than destroy pollutants, producing a spent material that requires careful disposal or regeneration (Fei et al., 2022; Boulkhessaim et al., 2022).

Membrane filtration, including nanofiltration (NF) and reverse osmosis (RO), can effectively separate POPs from water streams through size- and charge-based exclusion. However, these processes generate a concentrated brine or retentate stream laden with POPs, posing a secondary waste management challenge (Boulkhessaim et al., 2022).

#### Integrated and emerging hybrid technologies

The most promising advances come from integrating multiple mechanisms to overcome individual limitations. Bioelectrochemical systems (BES), such as microbial fuel cells, use electroactive bacteria to oxidize organic matter (including some POPs) at the anode, generating an electrical current. This system provides a permanent electron acceptor, which is particularly useful for stimulating the anaerobic degradation of pollutants, such as PAHs, in oxygen-limited environments (Feng et al., 2025). Bio-electro-Fenton (BEF) systems are a potent hybrid that combines BES with advanced oxidation. In a BEF system, bacteria at the anode generate electricity from organic matter. This electricity drives an electrochemical Fenton reaction at the cathode, producing hydroxyl radicals in situ to destroy POPs. A 2025 study using a nickel/cobalt MOF catalyst in a BEF cathode demonstrated 98% removal of the endocrine disruptor bisphenol-S at low concentrations while simultaneously generating electrical power. This exemplifies the potential for integrated systems to treat pollutants while recovering energy (Alinasab et al., 2025).

Table 5 summarizes the mechanisms, performance, and key considerations for the major technology classes discussed, providing a clear framework for comparison and selection.

**Table 5:** Comparative analysis of remediation technologies for POPs

Technology category	Specific method	Primary mechanism for POPs	Efficiency for POPs	Advantages	Major limitations and challenges
Chemical destruction	Persulfate-based AOPs (e.g., catalytic persulfate)	Radical (SO₄•⁻, •OH) and non-radical oxidation break C–Cl and C–C bonds	Removal of 85% or more for mixed POPs in leachate	High destruction efficiency; works on a wide range of POPs; wider pH range than Fenton	Potential toxic by-products; can require post-treatment; chemical/energy input
	Photocatalysis (e.g., metal oxide catalysts)	Light-generated radicals oxidize pollutants	High efficiency under optimized laboratory conditions	Utilizes (solar) light; can achieve mineralization	Catalyst recombination; limited visible-light activity; scaling challenges
Biological and bio-hybrid	Engineered microbial consortia (e.g., imGEMs with AOP pre-treatment)	Catalytic pre-treatment followed by enzymatic mineralization via engineered pathways	> 96.8% removal in integrated systems	Complete mineralization, sustainable, and lower long-term costs	Requires sophisticated bioengineering and system integration, with high complexity
	Microalgae–bacteria consortia	Synergistic metabolism; algae provide O_2_, bacteria degrade POPs	Enhanced vs. single-species; efficient for PCBs and PAHs	Eco-friendly; self-sustaining; low energy input	Optimization of consortia is complex and slower than chemical methods
	Phytoremediation with circular processing	Plant uptake and sequestration, followed by microbial fermentation of biomass	87–95% POP removal during fermentation of plant tissue	Very low cost, produces valuable fertilizer, and offers a circular solution	Very slow in situ; limited to root depth; requires biomass management
Physical separation	Nanoadsorption (e.g., CNTs, MOFs)	High-surface-area materials adsorb/concentrate POPs	High capacity for targeted POPs	Fast; tunable for specific POPs	Phase transfer only; creates concentrated waste; nanomaterial cost/risk
	Membrane filtration (NF/RO)	Physical size/charge exclusion separates POPs from water	High rejection rates for many POPs	Effective for water streams; produces clean permeate	High pressure/energy (RO); fouling; produces a concentrated waste stream
Integrated hybrid systems	Bio-electro-Fenton (BEF) systems	Microbial oxidation coupled with electro-Fenton destruction at the cathode	Up to 98% for specific POPs	Simultaneous treatment and energy recovery; in situ oxidant generation	System complexity, electrode/material costs, and optimization for diverse POPs
	Bioelectrochemical systems (BES)	Electrogenic bacteria oxidize organic matter, providing electrons for reduction or energy	Effective for PAHs; enhances anaerobic degradation	Generates energy; provides an electron acceptor for anaerobic zones	Lower power density; degradation rates for some POPs can be slow

Laboratory-scale remediation processes often face significant scalability challenges in field applications. Although high removal efficiencies are reported in controlled bioreactors, real landfill leachate exhibits extreme chemical complexity, with chemical oxygen demand (COD) ranging from 140 to 152,000 mg/L, reflecting a highly diverse pollutant profile (Chen et al., 2024; Wang & Qiao, 2024). Energy consumption varies substantially by technology. For landfill leachate treatment, AOPs such as ozonation can have a specific energy consumption as low as ~ 0.37 kilowatt-hours per cubic meter (kW·h/m^3^), whereas certain photo-Fenton processes can require up to ~ 39.2 kW·h/m^3^ (Koutsou et al., 2023). In contrast, a conventional activated sludge process for biological treatment was reported to consume about 0.6 kW·h/m^3^ (Koutsou et al., 2023). In terms of effectiveness, AOPs are recognized for achieving high levels of mineralization, with studies often targeting 50%, 75%, and 99% removal of total organic carbon (TOC) (Mousset et al., 2021). Specific applications to leachate have demonstrated TOC removal efficiencies ranging from 24.05% to 91.68% (Feng et al., 2025). Cost-effectiveness in continuous field applications remains a key hurdle, as selecting the optimal technology must balance operational costs, energy use, and environmental footprint (Wang & Qiao, 2024; Koutsou et al., 2023). Most critically, a persistent gap exists between innovative laboratory-validated technologies and commercially deployed systems. These interconnected limitations underscore the necessity for pilot-scale studies integrated with comprehensive life-cycle assessment (LCA), a standardized methodology (ISO 14040/44) for evaluating full environmental impacts before making robust technology recommendations.

### Machine learning in the treatment of POPs

A clear consensus emerges on the effectiveness of advanced ensemble algorithms (e.g., RF, XGBoost, Gradient Boosting) and neural networks in modeling the complex, nonlinear relationships inherent in these processes, underscoring a transformative role for ML in environmental treatment engineering. Beyond their role as “black box” predictors, these models serve as powerful diagnostic tools. Through interpretation techniques such as permutation variable importance (PVI), researchers can systematically decode the significance of operational parameters, consistently identifying factors like catalyst properties, pH, and reaction time as critical, and quantifying the influence of specific molecular structural features on degradability. This capability provides profound mechanistic insights that complement traditional experimental work. Ultimately, the integration of ML into this field is shifting the paradigm from purely empirical investigation to a data-driven, predictive science, enabling more efficient optimization of treatment processes, accurate forecasting of pollutant fate, and intelligent design of novel remediation technologies. Overall, these applications demonstrate that ML is not merely an add-on but is becoming integral to the engineering of next-generation remediation systems. By providing insights that transcend empirical observation, AI is helping to shift remediation from a predominantly empirical practice to a predictive science. This capability is crucial for developing intelligent, adaptive treatment architectures that address the dynamic and site-specific nature of POP contamination.

#### Predicting environmental fate and biodegradation

Cheng et al. addressed the significant challenge of predicting the anaerobic biodegradation half-lives of POPs in sediments and sludge, a process that is traditionally difficult and time consuming to measure experimentally (Cheng et al., 2024). Their comprehensive approach involved curating two large, high-quality datasets comprising 978 records for 206 distinct chemicals. They employed the LightGBM and Extra Tree algorithms to develop both binary classification models (to predict whether degradation is fast or slow) and regression models (to predict the specific half-life). The binary models achieved high accuracy of 81% for sediments and 80% for sludge. The regression models, while more challenging, provided quantitative predictions with *R*^2^ values of 0.56 and 0.31 for sediments and sludge, respectively. Their analysis underscored that both experimental conditions (e.g., temperature, inoculum dosage) and molecular structural features (e.g., the presence of aromatic rings and methyl groups) were critical determinants of degradation behavior. Practically, their models successfully predicted half-lives for 41% of sediment chemicals and 67% of sludge chemicals from a large inventory of over 4000 persistent, bioaccumulative, and toxic substances, demonstrating significant potential for environmental risk assessment (Cheng et al., 2024).

#### Machine learning in advanced oxidation processes

A substantial body of ML research has focused on optimizing AOPs, which are widely recognized for their high degradation efficiency but are characterized by complex, nonlinear reaction mechanisms. ML techniques offer a powerful means to capture these complexities and enhance process predictability and control.

Navidpour et al. (2024) investigated the photocatalytic degradation of PFOA using a data-driven ML approach. After a preliminary evaluation of seven algorithms, Adaptive Boosting (AdaBoost), GBM, and RF were selected for detailed modeling. Among these, the GBM model achieved the best predictive performance, with a coefficient of determination (*R*^2^) of 0.878. PVI analysis identified irradiation time as the most influential parameter governing PFOA degradation, followed by catalyst type and catalyst dosage. These findings underscore the ability of ensemble-based ML models to capture the dominant operational factors in photocatalytic AOP systems.

In a complementary study on PAH remediation in contaminated soils, Smara et al. (2024) evaluated the oxidative performance of hydrogen peroxide (H_2_O_2_) as a standalone oxidant and in combination with ferrous ions in the Fenton process (H_2_O_2_/Fe^2^⁺). Experiments used soil samples from a refinery site with an initial total PAH concentration of approximately 1200 mg kg⁻^1^. Systematic variation of oxidant dosages and molar ratios showed that H₂O₂ alone achieved degradation efficiencies of 57.0% to 75.6%. In contrast, the Fenton process significantly enhanced PAH removal, reaching efficiencies of 84.7% to 99.9% within 60 min. Kinetic analysis further showed that first-order rate constants increased with H_2_O_2_ dosage, from 0.013 min⁻^1^ to 0.023 min⁻^1^, while the Fenton process exhibited substantially higher reaction rates, reaching up to 0.066 min⁻^1^.

To complement the experimental findings, the authors integrated Gaussian Process Regression (GPR) to predict the reduced concentration ratio (*C*/*C*_0_) as a function of reaction time, H_2_O_2_ dosage, and Fe^2^⁺ concentration. Among 10 kernel functions evaluated, the Automatic Relevance Determination Exponential (ARD-Exponential) kernel with a constant basis function achieved optimal performance, yielding correlation coefficients (*R*) of 1.000 for the training dataset and 0.9997 for the validation dataset, along with exceptionally low RMSE values on the order of 10⁻^4^. Model performance and optimization were further enhanced using the Improved Grey Wolf Optimizer (IGWO) algorithm, which identified optimal operating conditions (60 min, 200 mmol H_2_O_2_, and 2 mmol Fe^2^⁺) that minimized the predicted *C*/*C*_0_ value. Under these conditions, the model predicted complete degradation (*C*/*C*_0_ = 0), in close agreement with the experimentally measured value of 0.001, corresponding to a PAH removal efficiency of 99.9% (Smara et al., 2024).

#### Machine learning in adsorption and filtration

Recent studies demonstrate the growing applicability of ML techniques for modeling and optimizing adsorption and filtration processes in water treatment. Akinpelu et al. (2023) developed a multivariate ML framework to predict the adsorptive removal efficiency of lindane from aqueous systems. The study compared an ANN with a conventional Stepwise Regression (SR) model. The ANN significantly outperformed SR, achieving coefficients of determination (*R*^2^) of 96.9% for the training dataset and 89.9% for the testing dataset, along with substantially lower error metrics, including RMSE and mean absolute percentage error (MAPE). These results highlight the superior predictive capability and robustness of ANN models for optimizing adsorption system performance.

In a related investigation, Karbassiyazdi et al. (2022) applied an eXtreme Gradient Boosting (XGBoost) framework to evaluate PFAS removal via adsorption. The model simultaneously assessed how adsorbent material properties and operational conditions influence removal efficiency. A dataset of 234 observations from 64 published studies (2008–2021) was used, with an 80/20 train–test split and Bayesian hyperparameter optimization. Model interpretability was enhanced using SHAP, which identified solution pH as the most influential predictor of the model’s output. Specifically, pH exhibited total gain values 3.19, 10.26, and 1.47 times greater than particle size for predicting adsorption capacity, equilibrium time, and removal efficiency, respectively. Resins (28.2%) and carbon-based adsorbents (35.9%) dominated the dataset and demonstrated the highest adsorption capacities, reaching up to 3067 mg g⁻^1^.

In contrast, parameters such as temperature and kinetic model order had relatively minor effects. The study further demonstrated that XGBoost, a scalable tree-based ensemble algorithm, is well suited to small, structured datasets (< 500 observations) commonly encountered in adsorption research, achieving area under the curve (AUC) values of 0.77 for equilibrium time, 0.51 for adsorption capacity, and 0.41 for removal efficiency. Collectively, these findings underscore the value of ML not only for process optimization but also for guiding the rational design of adsorbent materials for removing persistent contaminants (Karbassiyazdi et al., 2022).

Beyond adsorption, ML ensemble methods, including GBM, AdaBoost, and RF, have demonstrated strong predictive performance in modeling PFOS removal by nanofiltration. Among these, the optimized GBM model achieved exceptional accuracy, with an *R*^2^ of 0.975, explaining 97.5% of the variance in rejection efficiency, and a mean squared error (MSE) of 2.45. A comparative evaluation showed that the AdaBoost and RF models also performed robustly, with *R*^2^ values of 0.968 and 0.930, respectively. PVI analysis identified operating pressure and initial PFOS concentration as the most influential parameters, followed by membrane type and trivalent cation concentration. This integrated ML-based framework offers a powerful, data-driven approach to optimizing nanofiltration system design and operational strategies for the remediation of PFAS-contaminated water (Hosseinzadeh et al., 2022).

### Real-time process control and implementation challenges

While ML models demonstrate predictive capability, integrating them into operational remediation systems faces substantial implementation barriers. Model opacity poses regulatory hurdles; however, techniques like SHAP are being successfully integrated into hybrid ML frameworks, achieving over 94% fault detection accuracy in wastewater systems and helping to open the “black-box” for operator trust (Voipan et al., 2025). Computational performance varies widely, with advanced models, such as CNNs, demonstrating exceptional predictive accuracy (e.g., *R*^2^ = 0.999) in environmental management tasks (Chau et al., 2025).

Feedback control architectures are rapidly evolving beyond simple triggers. Multi-Agent Reinforcement Learning (MARL) frameworks mark a significant leap, demonstrating a 25.4% reduction in flooding volumes in an integrated urban drainage system while executing coordinated control decisions every 6.35 s (Huang et al., 2025). This highlights a shift toward adaptive, system-wide optimization, though challenges with sensor-actuator loop times persist.

Data security and standardization gaps are critical. IoT protocols like MQTT can implement Transport Layer Security/Secure Sockets Layer (TLS/SSL) encryption, but this security necessarily introduces overhead that reduces network throughput and can compromise measurement frequency. This risk is underscored by a reported 41% increase in weekly cyberattacks on IoT devices from 2022 to 2023 (Gentile et al., 2024). For global implementation, adopting FAIR (Findable, Accessible, Interoperable, Reusable) data principles is advocated to ensure interoperability (FAIR data principles, 2025). Practical tools like community-developed metadata reporting formats are emerging as essential for standardizing diverse environmental data, enabling data synthesis and federated learning (Crystal-Ornelas et al., 2022).

## From monitoring to action: barriers, economics, and long-term reliability

### Bridging monitoring and remediation: decision-support pathways

Translating continuous monitoring data into informed remediation decisions occurs in four interconnected stages, progressively transforming raw signals into actionable intelligence. The process begins with data acquisition and validation, where robust protocols and anomaly-detection algorithms distinguish genuine contamination events from sensor artifacts. The ALTEMIS project, for instance, demonstrates how ML can fuse multi-source sensing data to provide probabilistic, uncertainty-quantified early warnings, shifting the paradigm from reactive to proactive monitoring (Wainwright et al., 2022). This capability is further corroborated by physics-informed deep learning frameworks, which keep spatial errors within 20–22 m and achieve concentration *R*^2^ values of 0.76 even under significant monitoring noise (σ_h_ = 0.5–1 m), thereby supporting operator confidence in probability-based alerts over simplistic binary flags (Zhang et al., 2026).

Once validated, the data moves to threshold assessment and risk classification, where probabilistic risk classifiers replace rigid fixed standards that ignore site-specific heterogeneity and temporal variability. This approach is empirically validated by Tian et al., who employed Random Forest Regression (RFR) with 6,000 parameter iterations at a petrochemical site, achieving a Nash–Sutcliffe efficiency (NSE) > 0.96. Their study explicitly warns that deterministic models are unreliable under inherent hydrochemical variability, whereas probabilistic assessment effectively reduces the predicted threshold-exceedance probability for phenol from 64.0% to 15.7% under natural attenuation, thus enabling nuanced resource prioritization under uncertainty (Tian et al., 2024).

This probabilistic foundation feeds into predictive modeling and scenario evaluation, the third stage, which empowers operators to ask “what-if” questions before committing resources. Naghibi et al. propose an AI ensemble-based “what-if” engine that allows policymakers to quantitatively test alternative land-use and management scenarios and re-predict groundwater nitrate hotspot probabilities in real time (Naghibi et al., 2026). Furthermore, advanced surrogate models that integrate analytical solutions with U-Net architectures can complete a 12-year 3D groundwater simulation in just 600 ms on a single CPU core, a speed improvement of three orders of magnitude over numerical models, making rapid pump-and-treat (P&T) optimization feasible (Song et al., 2025). Complementing this, Wei et al. confirm that hybrid modeling approaches are robust at capturing complex variable interactions, directly supporting the shift from reactive to proactive management by testing interventions virtually and minimizing costly field trial-and-error (Wei et al., 2025).

Finally, remediation selection and adaptive control convert predictive insights into closed-loop action, with real-time sensor data continuously adjusting treatment parameters. Duan et al. developed an AI-based Self-Adaptive Dynamic Process Control (SADPC) system that integrates filtering, clustering, simulators, and a genetic algorithm-optimized model predictive control to effectively manage the dynamics and complexity of in situ bioremediation (Duan et al., 2024). For MOF-based POP removal, ML models optimized via covariance matrix adaptation achieve predictive accuracy with *R*^2^ exceeding 0.96, while SHAP analysis identifies key operational parameters (starting concentration, specific surface area, particle size) for real-time condition adjustment (Wei et al., 2026). The integration of PREC-Fenton with ANNs enables adaptive control for POP-B3 waste degradation, achieving 99% removal efficiency with *R*^2^ > 0.96 (Dienulloh et al., 2025). Concurrently, nano-biochar smart sensors coupled with AI algorithms enable closed-loop monitoring and adaptive control of treatment processes (Sharma & Kant, 2026), while data-driven digital twin frameworks for PFAS-selective resins dynamically optimize operational parameters and maintain closed-loop reusability (Luo, 2026).

For environmental managers, the cumulative benefit of this integrated pathway is enhanced operational intelligence. Validated data provides the foundation; probabilistic risk assessment guides prioritization; predictive modeling enables scenario testing; and adaptive control executes optimized responses. Crucially, this entire closed-loop continuum is conceptually framed by the “decision-grade digital twin” model, which must dynamically connect site conceptual models, constraint-bound predictions, uncertainty updates, and auditable trigger logic to meaningfully reduce ambiguity in intervention choices, operational intensity, and closure decisions (Cycoń, 2026). Nevertheless, as this critical review emphasizes, the persistent gap between laboratory-scale demonstrations and full field deployment serves as a sobering reminder that ongoing validation, standardized benchmarks, and regulatory acceptance remain essential prerequisites for translating these technological advances into routine practice (Cycoń, 2026).

### Pilot-scale validation and scalability

Conventional remediation strategies for POPs often show limited scalability and long-term effectiveness, a challenge that AI-enabled systems are increasingly poised to address (Bhat et al., 2026). For instance, a unified AI framework integrating Graph Neural Networks, Generative Adversarial Networks, and Reinforcement Learning has demonstrated computational scalability from 80 to 5,000 synthetic records (Rabbi, 2025). Similarly, the European REMEDYOS project is developing the first Integrated BioRemediation Platform (IBioRP), which leverages synergistic fungal–bacterial consortia, phytoremediation, and AI-driven digital tools (Climate-Resilient Integrated Bio- and Nature-Based Remediation of Pfas-Polluted Sites, 2026). The platform is explicitly designed for climate resilience, scalability, and adaptability. However, the path from laboratory success to field application is fraught with barriers, including complex pollutant interactions and the challenge of scaling technologies for real-world use (Bhat et al., 2026). To address this, the REMEDYOS project plans to validate its approach at a real pilot site at Technology Readiness Level 5 (TRL5) and replicate it in four additional European case studies (Climate-Resilient Integrated Bio- and Nature-Based Remediation of Pfas-Polluted Sites, 2026). Yet, the transition from controlled laboratory conditions to unpredictable field environments remains a major obstacle.

### Critical barriers to real-world implementation of AI-enabled POP management

Despite the transformative potential outlined in the preceding sections, widespread deployment of AI-enabled monitoring and remediation systems for POPs remains constrained by interconnected technical, methodological, infrastructural, and regulatory barriers. Translating promising laboratory and pilot-scale demonstrations into reliable operational systems requires not only accurate predictive models but also representative datasets, standardized analytical procedures, uncertainty-aware decision frameworks, interoperable sensor infrastructures, and sustained field validation. These requirements are especially important for POP management because reliable concentration measurements depend on rigorous sampling, analytical quality assurance, and interlaboratory comparability under the Stockholm Convention GMP (UNEP, 2026; UNEP, 2023; Magulova & Priceputu, 2016).

#### Data scarcity, quality, and labeling challenges

The performance of supervised machine-learning models depends strongly on the availability, representativeness, and quality of labeled training data. For POP monitoring, this is a substantial bottleneck because reliable concentration measurements require specialized sampling, extraction, instrumental analysis, quality-control procedures, and appropriately trained personnel. The UNEP GMP emphasizes the need for harmonized sampling and analytical approaches and underscores the importance of laboratory quality assurance and interlaboratory comparability for generating reliable POP concentration data (UNEP, 2023; Magulova & Priceputu, 2016). Consequently, large, consistently labeled, and directly comparable datasets suitable for AI model development remain scarce.

The limited availability and heterogeneity of environmental datasets also constrain the development of robust predictive models. A dataset of 978 experimental records covering 206 chemicals was used to model anaerobic biodegradation half-lives in sediment/soil and sludge. The resulting regression models achieved *R*^2^ values of 0.56 for sediment/soil and 0.31 for sludge, underscoring the difficulty of deriving robust predictive relationships from heterogeneous environmental and experimental data. Furthermore, the defined applicability domains yielded reasonable predictions for only 41% of more than 4,000 persistent, bioaccumulative, and toxic chemicals, underscoring the limitations of extrapolation beyond the available training domain (Cheng et al., 2024).

A related challenge is the lack of sufficiently standardized and interoperable datasets. The FAIR principles (Findable, Accessible, Interoperable, and Reusable) provide a framework for improving the discoverability and reuse of scientific datasets, but their implementation across environmental research infrastructures remains heterogeneous (Wilkinson et al., 2016; Nestola et al., 2026). For POP-focused AI development, future datasets should include standardized metadata describing sampling location, matrix, physicochemical conditions, analytical method, detection limits, quality-control procedures, temporal information, and uncertainty. Such harmonization would facilitate model training, independent validation, and cross-study comparisons. Federated learning may also enable model training across distributed datasets without requiring centralized transfer of raw data, offering a potential approach to addressing data fragmentation, privacy, and connectivity constraints (Miller et al., 2022).

#### Model overfitting, generalizability, and spatial transferability

High predictive performance in internal validation does not necessarily indicate that an AI model will remain reliable when deployed at a new location or under different environmental conditions. Environmental systems exhibit substantial spatial and temporal heterogeneity, while training datasets often represent only a limited number of sites, seasons, matrices, or contamination regimes. Consequently, random train–test splits can yield overly optimistic estimates when spatially or temporally correlated observations appear in both training and testing subsets. Geographically independent validation is therefore essential for determining whether model performance can be maintained under the environmental conditions encountered during real-world deployment (Ho et al., 2026; Tang et al., 2024).

This issue is particularly relevant to POPs because their environmental fate is governed by site-specific interactions among physicochemical properties, organic-carbon content, temperature, hydrology, sediment characteristics, biological activity, and transport processes. A model trained predominantly on data from one geographic region may therefore perform poorly when applied to another region with different environmental conditions. Spatial and domain shifts can substantially affect model performance, while transfer learning and domain-adaptation approaches offer potential pathways to improve extrapolation across locations (Ho et al., 2026; Poelzl et al., 2025; Ma et al., 2024). However, such approaches do not eliminate the need for geographically independent validation.

Physics-informed ML offers another promising strategy because it can incorporate known physical or environmental constraints into data-driven models. Physics-informed neural networks have shown promise for water contamination prediction and pollutant transport (Rashid et al., 2026; Rawden et al., 2026). Nevertheless, their application to POP monitoring remains in its early stages, and their capacity to improve spatial transferability requires validation with independent environmental datasets rather than relying solely on laboratory or simulation-based performance.

#### Uncertainty quantification and model opacity

For environmental decision-making, predictive accuracy alone is insufficient. AI-derived estimates should ideally be accompanied by quantitative information on the uncertainty associated with each prediction, particularly when model outputs may influence remediation priorities, regulatory decisions, or assessments of human and ecological exposure. However, uncertainty reporting remains inconsistent across environmental machine-learning applications. Only 22.5% of machine-learning datasets assessed in Earth-observation applications included any form of uncertainty information, and the reliability of the approaches used varied substantially (Singh et al., 2024).

Several approaches can address this limitation, including ensemble methods, Bayesian approaches, quantile regression, and conformal prediction. In particular, conformal prediction provides a model-agnostic framework for generating statistically valid prediction intervals or sets and can support distinguishing predictions at different confidence levels (Singh et al., 2024). For POP monitoring, uncertainty-aware predictions could enable AI systems to distinguish between high-confidence measurements and observations that require confirmation through conventional laboratory analysis.

Interpretability is a complementary challenge. Complex models, particularly deep neural networks, can make accurate predictions while offering limited insight into the relationships underlying those predictions. This “black-box” characteristic can reduce scientific interpretability and may complicate the adoption of AI outputs in high-stakes environmental decision-making. XAI approaches, including feature-attribution and local explanation methods, can provide additional information about the factors contributing to model predictions. XAI can therefore support model communication, diagnosis, and scientific interpretation, although limitations remain regarding explanation reliability, methodological consistency, and interpretation (Huang et al., 2024). Consequently, explainability should complement rather than replace independent model verification and validation.

#### Regulatory acceptance and standardization gaps

A major barrier to operational AI adoption is the lack of mature regulatory pathways for integrating AI-derived predictions into established POP monitoring and compliance frameworks. Under the Stockholm Convention, the GMP relies on reliable analytical measurements and extensive quality-assurance and interlaboratory-comparison procedures to produce comparable POP concentration data (UNEP, 2026; UNEP, 2023; Magulova & Priceputu, 2016). These established analytical requirements provide the reference framework against which emerging sensor- and AI-based measurements must be evaluated.

Accordingly, one of the central challenges is determining how AI-enabled measurements can be validated against established reference analytical methods and under what circumstances they can complement, rather than replace, conventional laboratory measurements. AI-generated concentration estimates from sensors may be particularly useful for screening, anomaly detection, prioritizing sampling locations, and early warning, whereas confirmatory regulatory determinations can continue to rely on validated analytical methods. Such a tiered monitoring framework could reduce the risk of prematurely substituting conventional analytical measurements.

Interoperability is another critical challenge. Environmental monitoring infrastructures increasingly integrate heterogeneous sensors, communication technologies, data formats, and computational platforms. IoT research has demonstrated the need for interoperability mechanisms that integrate protocols such as MQTT and CoAP with low-power communication technologies such as LoRa, while also highlighting trade-offs among interoperability, communication overhead, and energy consumption (Nguyen et al., 2018). For large-scale POP monitoring networks, standardized metadata, common data models, sensor calibration records, timestamps, geospatial information, detection limits, and uncertainty descriptors will therefore be essential for meaningful cross-platform integration.

Cybersecurity is an additional concern as monitoring systems become increasingly connected. IoT devices are highly vulnerable to cyberattacks, and an assessment of distributed IoT measurement systems reported a 41% increase in the average number of weekly attacks targeting IoT devices during the first 2 months of 2023 compared with 2022 (Gentile et al., 2024). Although this statistic is not specific to environmental monitoring, it underscores the broader cybersecurity risks associated with increasingly connected sensor infrastructures. In POP monitoring, compromise of sensor data, communication channels, or control systems could affect the integrity of contamination assessments and automated remediation decisions. Consequently, cybersecurity, authentication, data integrity, secure firmware management, and auditability should be treated as integral components of AI-enabled environmental monitoring rather than as secondary IT considerations.

#### Practical sensor deployment and edge computing constraints

The reliability of AI-enabled POP monitoring ultimately depends on the sensors that generate the underlying data. Although advanced chemical and biosensing technologies can provide sensitive, potentially continuous measurements, long-term field deployment remains challenging because environmental sensors are exposed to biofouling, matrix effects, sensor drift, calibration changes, physical degradation, and variable environmental conditions. These challenges are particularly important for aquatic monitoring. Biofouling is a major factor affecting the operation, maintenance, and data quality of submerged instrumentation, and estimates from the Alliance for Coastal Technologies indicate that biofouling-related costs can account for up to 50% of operational budgets, depending on location and season (Delgado et al., 2021). This figure should therefore be regarded as a context-dependent estimate rather than a universal proportion of operational costs.

Biofouling can shorten deployment intervals, increase maintenance requirements, cause sensor drift, reduce data quality, and shorten instrument lifetime (Delgado et al., 2021). The relatively short duration of many sensor-development evaluations, compared with the substantially longer timescales required for regulatory environmental monitoring, further underscores the need for long-term field validation. Long-term field trials under realistic contamination, temperature, salinity, turbidity, biological, and hydrological conditions are therefore required before AI-enabled sensors can be considered reliable components of continuous POP monitoring networks.

Computational constraints pose an additional challenge for autonomous monitoring stations. Advanced machine-learning models may exceed the memory, processing, energy, or thermal limits of remote edge devices. Edge computing can reduce communication requirements and latency by processing measurements locally before transmitting selected information to cloud infrastructure. Its application in water-quality monitoring demonstrates the potential value of local preprocessing and real-time prediction (Ren et al., 2022). However, resource-constrained devices require careful optimization of model size, memory footprint, computational complexity, and energy consumption.

Model compression, quantization, pruning, and lightweight architectures are promising approaches for enabling AI inference on edge devices. Nevertheless, optimization may involve trade-offs among predictive accuracy, computational efficiency, memory requirements, and robustness. For POP monitoring, evaluation of edge-enabled AI systems should therefore extend beyond predictive accuracy to include energy consumption, communication requirements, processing latency, sensor uptime, maintenance frequency, calibration stability, and failure rates under realistic field conditions.

The transition from laboratory-scale AI demonstrations to operational POP management therefore requires evaluation criteria that go beyond model accuracy. Robust systems must demonstrate data quality, external generalizability, uncertainty quantification, interpretability, analytical equivalence or complementarity with validated reference methods, cybersecurity, sensor stability, energy efficiency, maintainability, and long-term field reliability. A staged validation framework, progressing from laboratory testing to pilot-scale deployment, geographically independent validation, and ultimately long-term operational monitoring, would provide a credible pathway for integrating AI into POP monitoring and remediation while maintaining the analytical rigor and accountability required for environmental governance.

### Operational costs and energy considerations

The economic viability of these systems is a critical factor, and AI integration offers substantial cost-saving potential. ML and deep-learning models demonstrate high predictive accuracy (*R*^2^ > 0.99) for process optimization (Al-Jamimi et al., 2024), reducing operational costs and enhancing treatment performance. A case study of the PREC-Fenton system with ANN-based optimization reported a break-even point at 44% capacity, a net present value of USD 125,529, an internal rate of return of 33.48%, and a discounted payback period of 5 years (Dienulloh et al., 2025). Furthermore, AI-driven AOPs have demonstrated substantial energy and chemical savings, with studies reporting energy reductions of up to 20% in industrial wastewater treatment (de Matos Filho et al., 2026) and up to 50% in desalination applications (Alenezi & Alabaiadly, 2025), as well as processing cost reductions of 22–26% and chemical consumption reductions of 28–35% through deep learning–optimized Fenton-like reactions (Chen et al., 2025).

Energy consumption poses a dual challenge for AI-enabled POP remediation. On the one hand, AI optimization can significantly reduce the energy intensity of remediation processes; the PREC-Fenton system integrated with ANN optimization generated 300.17 kW with a conversion efficiency of 40% (Dienulloh et al., 2025). On the other hand, the environmental footprint of large-scale AI/ML infrastructure, including the high energy demands for model training and data center operations, is a significant concern. According to the International Energy Agency (IEA), data centers, AI, and cryptocurrency collectively consumed approximately 460 TWh of electricity globally in 2022, accounting for nearly 2% of global demand, a figure that may double by 2026 (Data Centres, Artificial Intelligence and Cryptocurrencies Eye Advanced Nuclear to Meet Growing Power Needs, 2024). These mitigation advantages are thus partially offset by the vast computational energy required to train such models, alongside the intensive resource extraction (Falk et al., 2026) and electronic waste generation associated with AI infrastructure (Nie et al., 2026).

### Long-term reliability and adaptive management

Looking ahead, long-term reliability remains a critical concern. For AI systems, issues such as data drift pose significant threats to long-term reliability as ML models enter production, particularly on resource-limited edge devices (Seo & Lim, 2026). The persistence of POPs, with their stable molecular structure and resistance to degradation, demands sustained performance over decades (Bhat et al., 2026). Future research must therefore focus on scaling technologies, improving cost-effectiveness, and integrating AI-powered smart monitoring systems for long-term environmental management. Achieving reliable long-term operation will require continuous monitoring of model performance, data quality, and system integrity, as well as the development of standardized protocols and multidisciplinary partnerships.

## Conclusion, recommendations, and future perspectives

POPs remain a major environmental challenge due to their persistence, long-range transport, bioaccumulation, and resistance to conventional degradation. This review shows that integrating advanced sensing, ML, IoT-enabled monitoring, and remediation technologies offers an important opportunity to strengthen POP management across detection, prediction, treatment, and environmental decision-making. The literature indicates that these technologies can improve the speed, continuity, and predictive capability of POP assessment while enabling more adaptive remediation. However, their current impact remains constrained by substantial differences in data quality, experimental conditions, model development, and validation practices.

A key finding of this review is that no single machine-learning approach is universally superior for POP monitoring or remediation. Model performance varies with dataset characteristics, environmental matrix, predictor structure, temporal dependence, prediction objective, and validation strategy. Consequently, reported performance metrics cannot be interpreted independently of the methodological context in which they were obtained. The literature also reveals a persistent gap between promising laboratory-scale results and reliable field application. Limited datasets, insufficient geographical diversity, inconsistent sensor calibration, heterogeneous benchmarking procedures, limited independent validation, and inadequate reporting of uncertainty continue to restrict reproducibility, transferability, and confidence in real-world deployment. These limitations indicate that future progress should focus not only on improving predictive accuracy but also on establishing reliable and comparable systems that can operate under complex environmental conditions.

Several emerging directions could address these limitations. Digital twins can provide dynamic virtual representations of contaminated environments and treatment processes, enabling the evaluation of monitoring strategies, process conditions, and remediation scenarios before field implementation. Physics-informed ML can incorporate established transport, reaction, and degradation principles into data-driven models, improving physical consistency and reducing reliance on large empirical datasets. Federated learning can leverage geographically distributed environmental data while keeping data within their originating institutions or monitoring networks, which could facilitate collaborative model development across jurisdictions. At the operational level, autonomous environmental monitoring systems that combine continuous sensing, adaptive sampling, robotics, and automated decision support could move POP assessment beyond periodic sampling toward responsive, site-specific monitoring. Edge AI could further support these systems by enabling local processing and rapid interpretation of sensor data where connectivity, bandwidth, or centralized computing resources are limited.

These developments, however, depend on a stronger foundation in data and validation. Establishing standardized, geographically diverse, FAIR environmental datasets covering POP occurrence, environmental conditions, sensor performance, and treatment outcomes should be a research priority. Equally important are harmonized procedures for sensor calibration, data preprocessing, model benchmarking, external validation, uncertainty characterization, and explainability. Such standards would enable meaningful comparisons across algorithms and studies, improve reproducibility, and provide a stronger basis for regulatory evaluation. Future research should therefore increasingly move from laboratory proof-of-concept studies toward independently validated pilot- and field-scale demonstrations that evaluate sensing, prediction, and remediation under realistic environmental conditions.

The evidence reviewed ultimately indicates that the future of POP management depends on integration rather than technological substitution. Advanced analytical methods, continuous monitoring, process-based knowledge, and remediation technologies should serve as complementary components of a validated environmental management framework. Establishing such integration, supported by standardized data, independent validation, physical consistency, and transparent uncertainty assessment, would provide a stronger scientific basis for shifting POP management from reactive detection and treatment to continuous, adaptive, and evidence-based environmental protection.

## Data Availability

No datasets were generated or analysed during the current study.
